# Nanostructured Aluminum Oxyhydroxide—A Prospective Support for Functional Porphyrin-Based Materials

**DOI:** 10.3390/ijms241512165

**Published:** 2023-07-29

**Authors:** Stepan M. Korobkov, Kirill P. Birin, Anatole N. Khodan, Oleg Yu. Grafov, Yulia G. Gorbunova, Aslan Yu. Tsivadze

**Affiliations:** 1 Frumkin Institute of Physical Chemistry and Electrochemistry, Russian Academy of Sciences, Leninsky pr., 31, bldg 4, 119071 Moscow, Russia; 2 Faculty of Chemistry, Lomonosov Moscow State University, GSP-1, 1-3 Leninskiye Gory, 119991 Moscow, Russia; 3 Kurnakov Institute of General and Inorganic Chemistry, Russian Academy of Sciences, Leninsky pr., 31, 119991 Moscow, Russia

**Keywords:** porphyrins, hybrid materials, nanostructured aluminum oxyhydroxide, imidazole, anchoring groups

## Abstract

A method for the grafting of unsymmetrical A_2_BC-type 5,15-bis(4-butoxyphenyl)-10-(4-carboxyphenyl)-20-(phenanthrenoimidazolyl)-porphyrin onto the surface of nanostructured aluminum oxyhydroxide modified with a single SiO_2_ layer (NAOM) was successfully developed. A straightforward procedure towards surface modification of NAOM allowed us to prepare a new porphyrin-containing hybrid material. The obtained 3D heterostructure was extensively characterized using XPS, TEM and diffuse reflectance spectroscopy. Structural and morphological peculiarities of the inorganic support before and after the immobilization procedure were studied and discussed in detail. The stability of the material against leaching and the porphyrin immobilization ratio ca. 14% by weight were also revealed.

## 1. Introduction

Porphyrin chemistry is a rapidly expanding field of research. These unique macrocyclic compounds were proven to be promising molecules in multiple application fields [1], namely photodynamic therapy [2,3,4], dye-sensitized solar cells [5,6], nonlinear optics [7,8], catalysis [9,10], etc. Various existing synthetic approaches [11,12,13,14,15] towards different structural types of tetrapyrrolic compounds have determined the enormous diversity of this class of molecules. In this respect, further achievements in porphyrin chemistry were attained with the development of porphyrin-based functional materials [16,17,18] in the past decade. The term “functional” is used to outline specific properties of the obtained material, such as luminescence [19], gas storage and separation [20,21], catalytic activity [22,23], etc. Progress in the development of hybrid functional materials is outlined in several comprehensive reviews [24,25,26,27]. Porphyrin chemistry is not outside this trend and recent advances in the development of porphyrin-based functional materials [10,23,28,29,30,31] has received growing attention in this field.

The development of porphyrin-based functional materials originates from the aim to utilize the outstanding physicochemical properties of these macrocyclic compounds in a convenient form. Notably, the majority of porphyrin-based functional materials belong to the family of covalent (COF) and metal–organic (MOF) frameworks [31,32,33,34]. However, the development of COFs and MOFs imposes some notable restrictions on the structure of the molecules and their functional groups. Consequently, it forces one to use only certain symmetrical types of porphyrins for the preparation of the abovementioned frameworks. It is possible to overcome this structural limitation with the immobilization strategy. This strategy implies surface modification of the inorganic support with porphyrin molecules bearing anchoring groups. In this case, porphyrin covers the surface of the inert supporting material, while in COFs and MOFs, porphyrin acts as a linker fragment and is distributed throughout the volume. In turn, the surface distribution is favored for the development of heterogeneous catalytic materials since the inner porphyrin molecules in the frameworks are typically less accessible for the reagents. Despite the advantages of the immobilization technique, the application of this method is still limited [18,23,35,36]. Nevertheless, a variety of anchoring groups are reported in literature [37] and some of them were comparatively analyzed [38,39]. Among them, the carboxylic group may serve well because of its stability and inertia.

**Chart 1:**
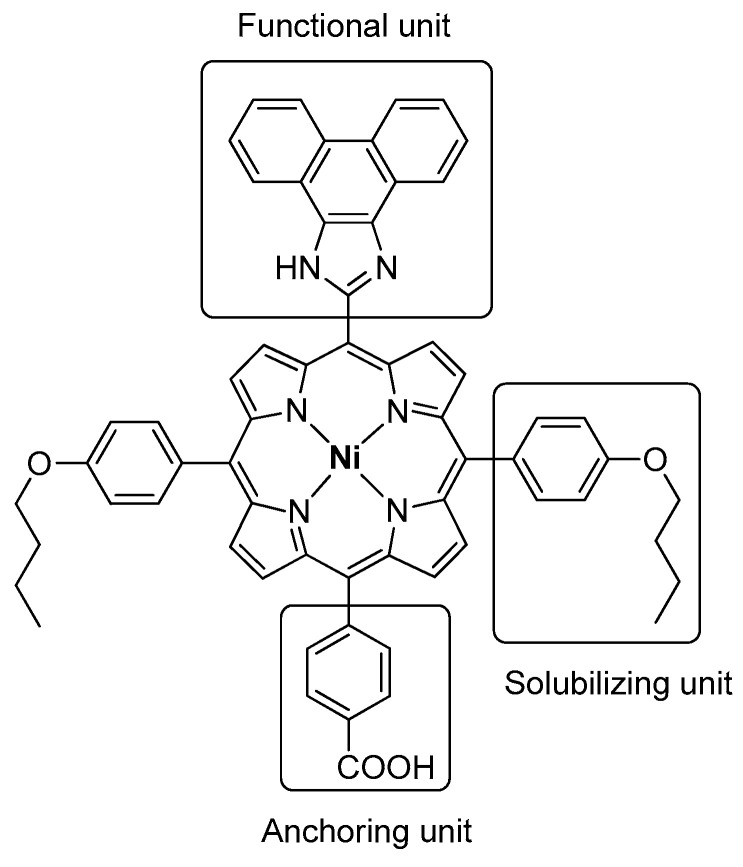
The substitution pattern of the porphyrin under investigation.

Herein, we report the first example of the surface modification of SiO_2_-modified nanostructured aluminum oxyhydroxide (NAOM) with porphyrin molecules. While different studies of typical tetraarylporphyrins on the surfaces of such materials as SiO_2_ and SiO_2_/ZnO are reported to date [40,41], the novelty of the present work consists both in the application of a scarcely studied fibrillar extremely porous Al_2_O_3_-based support and an unsymmetrical A_2_BC-type porphyrin, allowing further modification of the resulting hybrid material. For this purpose, an unsymmetrical porphyrin of A_2_BC type containing a bulky peripheral polyaromatic unit and an anchoring carboxy group at the opposite position of the macrocycle was designed (Chart 1). Nickel(II) as a metal center in the porphyrin macrocycle was selected in order to enhance the photostability of the molecule and to simplify the interpretation of the immobilization results. The opposite imidazophenanthrene unit was used as a model of a functional polyaromatic fragment with promising photophysical properties as well as a fragment closely related to a phenanthroline moiety. The 4-butoxyphenyl groups at *meso*-positions were used in order to enhance the solubility of the target molecule in organic media since the peripheral polyaromatic fragments have been previously found to decrease the solubility of porphyrins [42].

## 2. Results and Discussion

**Scheme 1:**
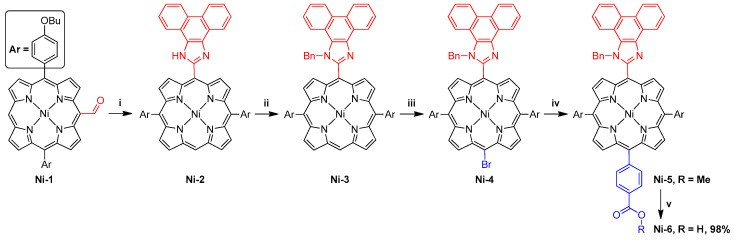
Synthesis of functionalized porphyrin **Ni-6**. *i*: phenanthrene-9,10-dione, NH_4_OAc, CHCl_3_/AcOH, reflux; *ii*: BnCl, DMF, K_2_CO_3_, 100 °C; *iii*: NBS, Py, CHCl_3_, −60 °C→r.t.; *iv*: 4-(methoxycarbonyl)phenylboronic acid, Pd(PPh_3_)_4_, Na_2_CO_3_, Tol/EtOH, reflux; *v*: NaOH, THF/H_2_O (12/1), reflux, 21 h.

The synthesis of the functionalized A_2_BC-type porphyrin began with the corresponding *meso*-formyl porphyrin (**Ni-1**), which was transformed into **Ni-5** in a multistep procedure (Scheme 1) [43]. The polyaromatic fragment was introduced to the porphyrin using the Debus–Radziszewski reaction of **Ni-1** with phenantrene-9,10-dione in the presence of an excess of ammonium acetate. The reaction product was alkylated with benzyl chloride to provide **Ni-3**. In turn, this product was subsequently brominated with NBS and applied to a Suzuki cross-coupling reaction with 4-methoxycarbonylphenylboronic acid in the presence of a Pd(Ph_3_P)_4_ catalyst. With the obtained porphyrin in hand, we proceeded to the hydrolysis stage which allowed the preparation of the target molecule **Ni-6** with almost quantitative yield. The porphyrin bearing an anchoring group was subsequently used in the immobilization stage. 

The photostability of the prepared **Ni-5** was tested by irradiation of its solutions with a 3W blue LED, namely the conditions of photobleaching of photoactive porphyrins reported previously [44,45]. Three different solvents were chosen (CCl_4_, toluene and CH_2_Cl_2_) for the tests, which differ in reported lifetimes of singlet oxygen. In contrast to photoactive compounds, Ni-5 showed less than 1% degradation in all cases, detected by decreases in the Soret band in UV–Vis spectra of solutions. It should be mentioned that the observed variation in Soret band absorption is comparable with the baseline drift in spectra and, thus, could not be severely analyzed.

Nanostructured aluminum oxyhydroxide (NAO) is a low-density porous material with fibrillar structure. Its chemical formula can be designated as Al_2_O_3_×*n*H_2_O, where *n* ~3.6. The NAO samples were grown in climatic chambers at 25 °C in air atmosphere with 70–75% humidity by the surface oxidation of a thin liquid alloy Hg(Al) layer deposited on an aluminum plate [46]. Treatment of NAO samples with saturated vapor of methyltrimetoxysilane (MTMOS) at room temperature led to hydrolysis and the formation of chemically adsorbed groups on the surface: [=Al–O–]Si–(CH_3_)|surf] [47]. Further annealing of the sample at 1000 °C allowed us to obtain a NAOM sample with a SiO_2_ layer of ca. single monolayer thickness. It should also be mentioned that the selected annealing temperature is the optimal balance between the enhancement of the mechanical durability and the unavoidable decrease in the porosity of the material [48,49]. Previously, it was shown that immobilization of a crown-substituted phthalocyanine molecule on the surface of NAOM is characterized by stronger binding compared to its non-modified analogue [47], while the annealing stage is essential in improving mechanical durability of the inorganic support in order to simplify its handling.

**Figure 1:**
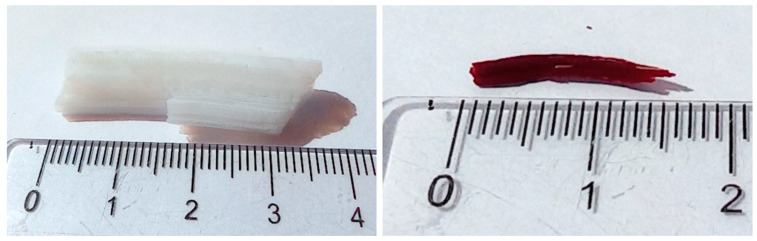
Prepared NAOM sample (**left**) and a fragment of a hybrid material (**right**).

The immobilization was performed by treatment of the annealed NAOM sample with the solution of **Ni-6** in CH_2_Cl_2_. The prolonged exposure of NAOM in the solution containing **Ni-6** resulted in a drastic change in color of the material from white to deep red (Figure 1) while the solution was considerably decolorized. The prepared material was repeatedly washed with CH_2_Cl_2_ in 24 h intervals until leaching was not observed and the washings were colorless. The analysis of the washings with UV–Vis allowed us to evaluate the amount of recovered **Ni-6** and, thus, to analyze the ratio of porphyrin in the prepared hybrid material, which was found to be ca. 14% by weight. The prolonged exposure of the obtained material in CH_2_Cl_2_ did not reveal further leaching of the porphyrin.

Along the immobilization, the tendency of the monolithic NAOM sample to fall into fibrillary pieces and powder-like particles was observed. This behavior of NAO and NAOM materials could be reasonably attributed to their low mechanical strength which is insufficient to survive the surface tension and capillary effects of the solvents. The observed behavior of the material is in consistency with previously reported peculiarities [47].

**Figure 2:**
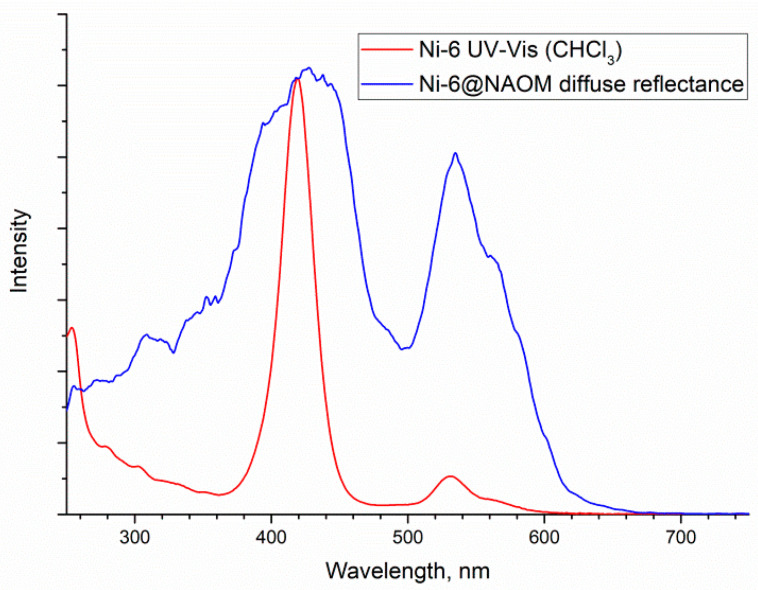
Normalized UV–Vis spectrum of **Ni-6** in CHCl_3_ solution and diffuse reflectance spectrum of **Ni-6@NAOM**.

The prepared hybrid **Ni-6@NAOM** material was characterized by a set of applicable physicochemical methods. First, the diffuse reflectance spectrum was acquired for the prepared material using an integrating sphere (Figure 2). The Soret band at 432 nm and Q bands at 543 and 576 nm were observed in the UV–Vis spectrum of **Ni-6** in CHCl_3_ solution. In turn, the diffuse reflectance spectrum of **Ni-6@NAOM** demonstrates a similar set of bands with a significant broadening in comparison with the spectrum in solution. Such broadening is typical for solid materials. The absence of any considerable shifts of bands within the spectra upon immobilization allows us to assume the absence of any specific interactions between the porphyrin molecules at the surface of NAOM and, thus, its monomeric state.

X-ray photoelectron spectroscopy (XPS) was used to characterize the surface of the hybrid material. This method was widely used previously for the characterization of a variety of free-base porphyrins and metal complexes [50,51,52], including Ni(II) porphyrinates [53,54]. Grafting of the free-base and Cu(II) porphyrinates to TiO_2_ (anatase) with subsequent characterization by means of XPS can also also found in literature [55]. The prepared hybrid material is inhomogeneous; therefore, quantitative analysis based on peak intensities may lead to overestimation of porphyrin loading [56]. In this study, we use XPS to prove the elemental composition of the hybrid material and to observe changes in the composition of the inorganic support after each modification procedure.

**Figure 3:**
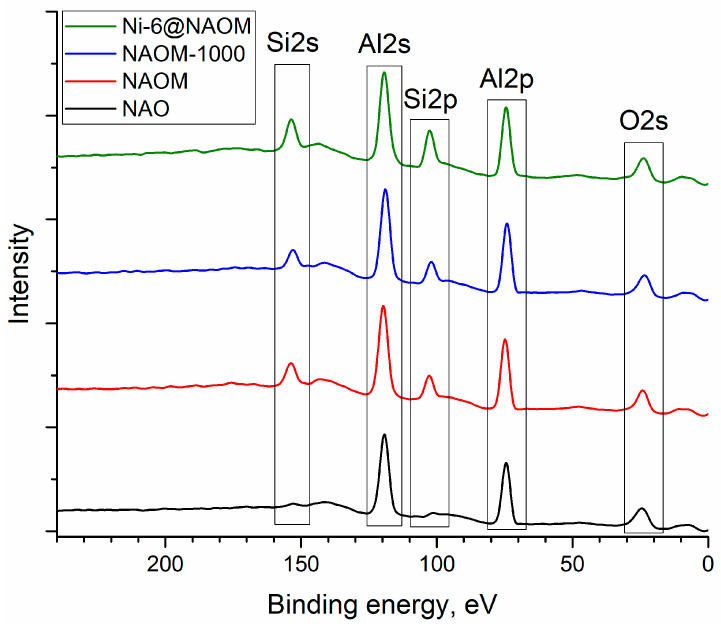
Stacked XPS spectra (low binding energy region).

XPS spectra were recorded for raw NAO samples before and after modification with MTMOS, after annealing at 1000 °C and after immobilization of **Ni-6**. Changes in the composition of the inorganic support during the abovementioned procedures could be observed in the low-binding-energy region (Figure 3). Treatment of the NAO samples with MTMOS expectedly led to the appearance of Si2s and Si2p peaks in the XPS spectrum of NAOM. Annealing at 1000 °C of the NAOM sample and further immobilization of porphyrin **Ni-6** did not result in any notable changes in the mentioned XPS region. Indeed, silicon and aluminum ratios did not reveal considerable changes after the modification procedures. The O2s peak remained virtually unchanged after the annealing stage despite the removal of the adsorbed water. As was noted above, surface inhomogeneity of the material complicates quantitative analysis of the sample; therefore, peak intensities cannot provide reliable information about the composition of the material.

**Figure 4:**
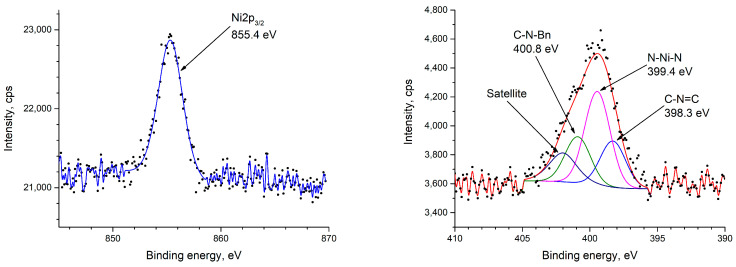
Ni2p_3/2_ peak (**left**) and deconvolution of N1s peak (**right**) in XPS spectrum of **Ni-6@NAOM**.

The expected Ni2p_3/2_ peak was observed in the XPS spectrum of **Ni-6@NAOM**, confirming the successful immobilization of the porphyrin on the surface of NAOM along with the abovementioned diffuse reflectance spectrum (Figure 4). The binding energy of the Ni2p_3/2_ peak is in agreement with the reported data [52]. The N1s peak in the XPS spectrum of **Ni-6@NAOM** was successfully deconvoluted into four peaks, corresponding to a pyrrolic fragment (C-N=C, 398.3 eV), coordination center (N-Ni-N, 399.4 eV), benzylic fragment (C-N-Bn, 400.8 eV) and shake-up satellite (401.9 eV). Unfortunately, the deconvolution of the C1s peak could not be performed since hydrocarbon contaminants were present in NAOM sample before the immobilization procedure.

**Figure 5:**
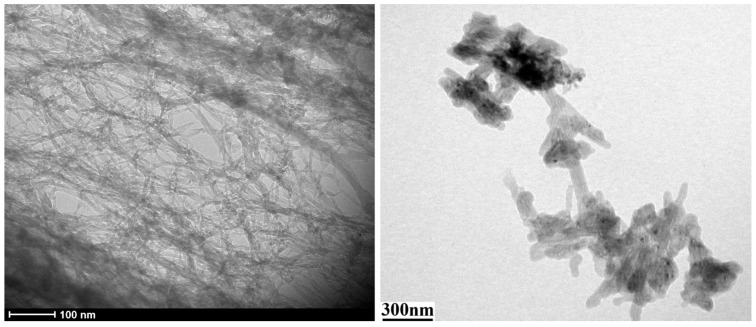
TEM images of raw NAO sample (**left**) and **Ni-6@NAOM** (**right**).

Figure 5 shows TEM images of a NAO sample and the **Ni-6@NAOM** hybrid. The initial NAO sample can be described as a porous material consisting of a 3D grid of interweaved fibrils. After all modification procedures, the porous material notably changed its morphology. Annealing at 1000 °C caused shrinkage of fibrils along with the beginning of crystallization of amorphous alumina to θ-Al_2_O_3_ [46]. These processes are the main contributors to the changes in the morphology.

## 3. Materials and Methods

All used reagent-grade chemicals were purchased from commercial suppliers, unless otherwise stated. The solvents have been purified according to conventional methods [57].

MALDI TOF mass spectra were recorded using a Ultraflex spectrometer (Bruker Daltonics, Switzerland) in positive ions mode using 2,4,6-Trihydroxyacetophenone (THAP) as a matrix. UV–Vis spectra were recorded using an Evolution 200 spectrophotometer (Thermo Scientific, MA USA) in rectangular quartz cells with a 0.1–10 mm optical path or with ISA-220 diffuse reflectance accessory in a 250–900 nm range. NMR spectra were recorded using a Avance III (Bruker, Fällanden, Switzerland) spectrometer with 600.13 MHz proton frequency in CDCl_3_ at ambient temperature with the use of the residual solvent signal as an internal reference.

The morphology of NAO samples and the porphyrin-containing hybrid was examined using the TEM method on a JEM 1210 electron microscope (JEOL, Tokyo, Japan).

X-ray photoelectron spectroscopy (XPS) studies were performed using an ESCA+ spectrometer (OMICRON, Berlin, Germany) with an aluminum anode equipped with an AlK_α_ XM1000 monochromatic X-ray source (with an emission energy of 1486.6 eV and a power of 252 W). The pressure in the analyzer chamber was kept no higher than 8 × 10^−10^ mbar and the analyzer’s pass energy was 20 eV. To allow for sample charging, the position of XPS peaks was standardized relative to the C 1s peak of hydrocarbon contaminants (adsorbed impurities from the atmosphere), the bonding energy of which was assumed to be 285.0 eV. The spectra were decomposed using the UNIFIT 2009 program after subtracting a background determined according to Shirley [58]. Peak positions were determined with an accuracy of ±0.1 eV.

The growth of the aluminum oxyhydroxide was performed in a WK3-180/40 climatic chamber (Weiss Umwelttechnik GmbH, Reiskirchen, Germany).

Chromatographic purification was performed using Macherey-Nagel Silica 60, 0.063–0.2 mm. Merck aluminum plates (TLC Silica 60 F254) were used for TLC analysis with hexane/CH_2_Cl_2_ and CH_2_Cl_2_/MeOH mixtures as eluents. Gel permeation chromatography was performed with Bio-Beads SX-1 sorbent (Bio-Rad, Hercules, CA, USA) in a CHCl_3_:MeOH (98:2) mixture.

Nickel(II) 5-formyl-10,20-di(4-butoxyphenyl)porphyrin **Ni-1** was prepared following the reported procedure [59]. Porphyrins **Ni-2**–**Ni-5** were prepared from **Ni-1** as reported previously [43,60]. The detailed synthetic procedures and spectral characteristics of all compounds used in the study (Figures S1–S24) can be found in ESI.

**[5-benzylimidazophenantrene-10,20-di(4-butoxyphenyl)-15-(4-carboxyphenyl)porphyrinato]nickel(II) Ni-6**. **Ni-5** (0.047 mmol, 52 mg) was dissolved in THF (25.2 mL). Sodium hydroxide (4.7 mmol, 188 mg) was dissolved in water (2.1 mL) and added to the THF solution. The mixture was refluxed for 21 h upon stirring and then quenched with HCl (38%, 570 μL). Then, CHCl_3_ (100 mL) and water (100 mL) were added. The organic layer was separated and evaporated to dryness. The solid residue was dissolved in CH_2_Cl_2_ and applied to a short plug of silica packed in CH_2_Cl_2_. A mixture of CH_2_Cl_2_ and MeOH (0→5% of MeOH) was further used as eluent. The obtained fraction was purified with size-exclusion chromatography producing 98% (50 mg) of pure compound.

^1^H NMR (600 MHz; CDCl_3_/MeOD (85/15); δ, ppm; *J*, Hz): 9.17 (d, 1H, ^3^*J =* 7.9, H_Phen_), 8.93 (d, 1H, ^3^*J =* 8.7, H_Phen_), 8.88–8.84 (m, 3H: 2H_β_, H_Phen_) 8.79 (d, 2H, ^3^*J =* 4.9, H_β_), 8.75 (d, 2H, ^3^*J =* 4.9, H_β_), 8.57 (d, 2H, ^3^*J =* 4.9, H_β_), 8.20–8.11 (br m, 3H: 2H_Carboxy-Ph_, H_Phen_), 8.07–7.67 (br m, 4H, H_Ar_), 7.85 (t, 1H, ^3^*J =* 7.5, H_Phen_), 7.77 (t, 1H, ^3^*J =* 7.8, H_Phen_), 7.62 (t, 1H, ^3^*J =* 7.8, H_Phen_), 7.46 (t, 1H, ^3^*J =* 7.6, H_Phen_), 7.18 (d, 4H, ^3^*J =* 7.7, H_Ar_), 6.98–6.89 (m, 3H, H_Bn_), 6.73 (d, 2H, ^3^*J =* 7.4, H_Bn_), 5.51 (s, 2H, H_Bn_), 4.17 (t, 4H, ^3^*J =* 6.5, H_O-Bu_), 1.92 (quint, 4H, ^3^*J =* 6.8, H_O-Bu_), 1.62 (sext, 4H, ^3^*J =* 7.4, H_O-Bu_), 1.07 (t, 6H, ^3^*J =* 7.4, H_O-Bu_).

MALDI TOF MS: m/z calculated for C_69_H_54_N_6_NiO_4_ 1088.36, found 1089.38 (MH^+^).

UV–Vis (CHCl_3_; λ_max_/nm; log ε): 268 (4.80), 292sh (4.35), 316sh (4.21), 432 (5.18), 543 (4.12), 576 (3.70).

**Preparation of modified nanostructured aluminum oxyhydroxide (NAOM).** The procedures for the preparation of nanostructured aluminum oxyhydroxide (NAO) and its further modification were similar to those previously reported [46,47,61]. A 10 × 10 mm high-purity aluminum plate (A-5N aluminum) was rinsed with isopropanol and the surface of the plate was treated with 2 M KOH solution for 10 min. Afterwards, the plate was treated with 2 M HNO_3_ solution containing 0.1 M Hg(NO_3_)_2_ and 0.05 M AgNO_3_ for 10 min. The plate was subsequently dried, fixed in a holder and placed on a cooler (20 °C) in the climatic chamber (70% humidity, 25 °C). The growth rate was ca. 1 cm/h. The grown fibrillar monolith (95 mg) was further used for the preparation of modified NAO (NAOM). NAO samples were placed in a desiccator at ambient temperature and treated with saturated vapor of methyltrimethoxysilane (MTMOS). After 4 h, the NAOM samples (105 mg) were removed from the desiccator. The samples were annealed at 1000 °C for 4 h so the resulting mass of the samples was 61 mg.

**Immobilization of Ni-6**. Porphyrin **Ni-6** (10 mg) was dissolved in CH_2_Cl_2_ (10 mL) and added to the annealed NAOM sample (58 mg). After 10 min of stirring, the solid material was centrifuged and washed with 30 mL of CH_2_Cl_2_. The centrifugation and washing procedure was repeated 5 times with 24 h intervals between each procedure. Liquid phase was removed with a pipette and the solid material was left at ambient temperature to remove residual solvent.

## 4. Conclusions

The present work demonstrates the first example of the surface modification of NAOM with a porphyrin bearing an anchoring group, providing a hybrid material with a porphyrin ratio ca. 14% by weight. The prepared novel-type hybrid material was characterized with a set of physicochemical methods to obtain insight into its composition and morphology. Thus, diffuse reflectance spectroscopy along with XPS served as reliable proof of the composition of the hybrid material while transmission electron microscopy demonstrated the evolution of the morphology of the porous material upon modification procedures. Concluding, this research forms the basis for the upcoming development of a new generation of macroscopic porphyrin-based hybrid materials for further application in heterogeneous catalysis.

## Data Availability

Data is contained within the article or Supplementary Material.

## Supplementary Materials

The following supporting information can be downloaded at: https://www.mdpi.com/article/10.3390/ijms241512165/s1. Reference [60] is cited in the supplementary materials.
