# Supplementary material for: Nanostructured Aluminum Oxyhydroxide—A Prospective Support for Functional Porphyrin-Based Materials
Source: Int J Mol Sci. 2023 Jul 29;24(15):12165. doi: 10.3390/ijms241512165 (PMC10418628; doi:10.3390/ijms241512165)
Supplement: Supplementary file 1 [file ijms-24-12165-s001.zip › ijms-2492705-supplementary.pdf]

SUPPORTING INFORMATION

for

Nanostructured aluminum oxyhydroxide - a prospective support for functional porphyrin-based materials

Stepan M. Korobkov[1,2], Kirill P. Birin[1,*], Anatole N. Khodan[1], Oleg Yu. Grafov[1], Yulia G. Gorbunova[1,3], Aslan Yu. Tsivadze[1,3]

[1] Frumkin Institute of Physical Chemistry and Electrochemistry RAS, 119071, Leninsky pr., 31, bldg 4, Moscow, Russia

[2] Lomonosov Moscow State University, Faculty of Chemistry, 119991, GSP-1, 1-3 Leninskiye Gory, Moscow, Russia

[3] Kurnakov Institute of General and Inorganic Chemistry RAS, 119991, Leninsky pr., 31, Moscow, Russia

Contents

General remarks 2

Synthetic procedures and characterization 2

Spectral data of Ni-3 4

Spectral data of Ni-4 7

Spectral data of Ni-5 10

Spectral data of Ni-6 13

General remarks

Phenantrenedione [1], **2H-1**, **Ni-1** [2] were prepared following to published procedures.

Synthetic procedures and characterization

[5,15-di-(4-butoxyphenyl)-10-imidazophenantreneporphyrinato]nickel(II) Ni-2. Mixture of **Ni-1** (0.30 mmol, 200 mg), phenantrene-9,10-dione (0.60 mmol, 125 mg) and NH_4OAc (6 mmol, 460 mg) in $CHCl_3$ (51 ml) and AcOH (9 ml) was gently refluxed for 7 hours. The same amount of phenantrene-9,10-dione and NH_4OAc was added and reflux was continued for 15 hours. The mixture was cooled to ambient temperature, diluted with $CHCl_3$ (50 ml) and extracted with water (60 ml). Organic layer was separated, evaporated to dryness and applied in CH_2Cl_2 to a silica column packed in hexane/CH_2Cl_2 (50→100% of CH_2Cl_2) and in CH_2Cl_2/MeOH (0.2→1% of MeOH) mixtures. The target product was purified from admixtures by size-exclusion chromatography. The separation provided 59% (156 mg) of pure product.

MALDI TOF MS: m/z calculated for $C_{55}H_{44}N_6NiO_2$ 878.29, found 878.23.

UV-Vis ($CHCl_3$; λ_{max}, nm; log ε): 251 (4.71), 275sh (4.31), 304sh (4.13), 414 (5.04), 525 (4.05), 554 (3.74).

[5-benzylimidazophenantrene-10,20-di-(4-butoxyphenyl)porphyrinato]nickel(II) Ni-3. BnCl (34 μl) and K_2CO_3 (0.349 mmol, 48 mg) were added to the solution of **Ni-2** (0.058 mmol, 51 mg) in DMF (7.5 ml) under argon atmosphere. The mixture was stirred at 110 °C for 20 hours. The solution was diluted with ethyl acetate (50 ml) and extracted with water (3×50 ml), then evaporated to dryness and applied in CH_2Cl_2 to silica column packed in hexane/CH_2Cl_2 (50→100% of CH_2Cl_2) mixture. The obtained fraction was evaporated and purified by size-exclusion chromatography providing 88% (49 mg) of pure compound.

MALDI TOF MS: m/z calculated for $C_{62}H_{50}N_6NiO_2$ 968.33, found 968.49.

^1H NMR (600 MHz; $CDCl_3$; δ, ppm; J, Hz): 9.80 (s, 1H, H_{meso}), 9.16 (d, 1H, ^3J = 7.9, H_{phen}), 9.08 (d, 2H, ^3J = 4.7, $H_β$), 8.94 - 8.88 (m, 5H: 4$H_β$, 1H_{Phen}), 8.85 (d, 1H, ^3J = 8.7, H_{phen}), 8.83 (d, 2H, ^3J = 4.9, $H_β$), 8.18 – 7.55 (br s, 4H, H_{Ar}), 8.11 (d, 1H, ^3J = 8.3, H_{phen}), 7.84 (t, 1H, ^3J = 7.4, H_{phen}), 7.76 (t, 1H, ^3J = 7.8, H_{phen}), 7.60 (t, 1H, ^3J = 7.8, H_{phen}), 7.44 (t, 1H, ^3J = 7.6, H_{phen}), 7.22 (d, 4H, ^3J = 8.0, H_{Ar}), 6.94 – 6.85 (m, 3H: 1H_{Bn}, 2H_{Bn}), 6.68 (d, 2H, ^3J = 7.6, H_{Bn}), 5.42 (s, 2H, H_{Bn}), 4.21 (t, 4H, ^3J = 6.5, $H_{O\text{-}Bu}$), 1.95 (quintet, 4H, ^3J = 6.6, $H_{O\text{-}Bu}$), 1.66 (sextet, 4H, ^3J = 7.4, $H_{O\text{-}Bu}$), 1.10 (t, 6H, ^3J = 7.4, $H_{O\text{-}Bu}$).

UV-Vis ($CHCl_3$; λ_{max}, nm; log ε): 254 (4.88), 279sh (4.42), 303sh (4.26), 411 (5.25), 524 (4.19), 553 (3.81).

[5-benzylimidazophenantrene-15-bromo-10,20-di-(4-butoxyphenyl)porphyrinato]nickel(II) Ni-4. Porphyrin **Ni-3** (0.051 mmol, 49 mg) was dissolved in $CHCl_3$ (9.8 ml). The solution was cooled to -60 °C, then pyridine (98 μl) and NBS (0.051 mmol, 9 mg) were added under argon atmosphere. The mixture was stirred for 0.5 hours without additional heating or cooling, the temperature raised to -5°C, then acetone (1 ml) was added. The obtained mixture was evaporated to dryness and applied in CH_2Cl_2 to a silica column packed in hexane/CH_2Cl_2 (50→75% of CH_2Cl_2) mixture. The obtained fraction provided 94% (50 mg) of product.

MALDI TOF MS: m/z calculated for $C_{62}H_{49}BrN_6NiO_2$ 1046.25, found 1046.07.

^1H NMR (600 MHz; $CDCl_3$; δ, ppm; J, Hz): 9.48 (d, 2H, ^3J = 5.0, $H_β$), 9.14 (d, 1H, ^3J = 7.9, H_{phen}), 8.90 (d, 1H, ^3J = 8.6, H_{phen}), 8.86 - 8.81 (m, 3H: 2$H_β$, 1H_{phen}), 8.79 (d, 2H, ^3J = 4.9, $H_β$), 8.72 (d, 2H, ^3J = 5.0, $H_β$), 8.08 (d, 1H, ^3J = 8.3, H_{phen}), 8.05 - 7.63 (br s, 4H, H_{Ar}), 7.83 (t, 1H, ^3J = 7.4, H_{phen}), 7.75 (t, 1H, ^3J = 7.8, H_{phen}), 7.59 (t, 1H, ^3J = 7.8, H_{phen}), 7.43 (t, 1H, ^3J = 7.6, H_{phen}), 7.19 (d, 4H, H_{Ar}), 6.95 - 6.83 (m, 3H, H_{Bn}), 6.66 (d, 2H, ^3J = 7.6, H_{Bn}), 5.37 (s, 2H, H_{Bn}), 4.20 (t, 4H, ^3J = 6.5, $H_{O\text{-}Bu}$), 1.93 (quintet, 4H, ^3J = 7.01, $H_{O\text{-}Bu}$), 1.64 (sextet, ^3J = 7.4, 4H, $H_{O\text{-}Bu}$), 1.09 (t, 6H, ^3J = 7.4, $H_{O\text{-}Bu}$).

UV-Vis ($CHCl_3$; λ_{max}, nm; log ε): 254 (4.88), 279sh (4.41), 302sh (4.30), 421 (5.25), 534 (4.19), 568 (3.80).

[5-benzylimidazophenantrene-15-(4-(methylcarboxy)phenyl)-10,20-di(4-butoxyphenyl)porphyrinato]nickel(II) Ni-5. Porphyrin **Ni-4a** (0.028 mmol, 29 mg) was dissolved in toluene (13.3 ml) and EtOH (2.7 ml) mixture, then Na_2CO_3 (0.280 mmol, 30 mg) and 4-(Methoxycarbonyl)benzeneboronic acid (0.140 mmol, 23 mg) were added. Finally, $Pd(PPh_3)_4$ (7.784 μmol, 9 mg) was added to the mixture under argon atmosphere. The solution was refluxed for 20 hours with stirring. The obtained mixture was evaporated and applied in CH_2Cl_2 through a silica column using hexane/CH_2Cl_2 (0→100% of CH_2Cl_2) and CH_2Cl_2/MeOH (0.2→2% of MeOH) mixtures as eluent. The residue was evaporated and purified by size-exclusion chromatography providing 90% (28 mg) of pure compound.

MALDI TOF MS: m/z calculated for $C_{70}H_{56}N_6NiO_4$ 1102.37, found 1102.32.

^{1}H NMR (600 MHz; $CDCl_3$; δ, ppm; J, Hz): 9.15 (d, 1H, ^3J = 7.9, H_{Phen}), 8.92 (d, 1H, ^3J = 8.6, H_{Phen}), 8.88 (d, 2H, ^3J = 5.0, $H_β$), 8.85 (d, 1H, ^3J = 8.6, H_{Phen}), 8.80 (m, 4H, $H_β$), 8.69 (d, 2H, ^3J = 4.9, $H_β$), 8.38 (d, 2H, ^3J = 7.7, $H_{Carboxy-Ph}$), 8.30 - 7.68 (br m, 6H: 4H_{Ar}, 2$H_{Carboxy-Ph}$), 8.11 (d, 1H, ^3J = 8.3, H_{Phen}), 7.84 (t, 1H, ^3J = 7.5, H_{Phen}), 7.76 (t, 1H, ^3J = 7.8, H_{Phen}), 7.61 (t, 1H, ^3J = 7.8, H_{Phen}), 7.45 (t, 1H, ^3J = 7.6, H_{Phen}), 7.21 (d, 4H, ^3J = 8.2, H_{Ar}), 6.96 - 6.86 (m, 3H, H_{Bn}), 6.69 (d, 2H, ^3J = 7.4, H_{Bn}), 5.41 (s, 2H, H_{Bn}), 4.20 (t, 4H, ^3J = 6.5, H_{O-Bu}), 4.09 (s, 3$H_{Carboxy-Me}$), 1.93 (quintet, 4H, ^3J = 7.0, H_{O-Bu}), 1.64 (sextet, 4H, ^3J = 7.5, H_{O-Bu}), 1.08 (t, 6H, ^3J = 7.4, H_{O-Bu}).

UV-Vis ($CHCl_3$; λ_{max}, nm; log ε): 254 (4.4), 280 (3.94), 303 (3.81), 419 (4.77), 530 (3.71), 564 (3.27).

[5-benzylimidazophenantrene-15-(4-(carboxy)phenyl)-10,20-di(4-butoxyphenyl)porphyrinato]nickel(II) Ni-6. **Ni-5** (0.011 mmol, 12 mg) was dissolved in THF (5.8 ml). NaOH (44 mg, 1.1 mmol) was dissolved in water (0.50 ml) and added to the THF solution. The mixture was refluxed for 30 hours upon stirring and then quenched with HCl (38%, 50 μl) and H_2O (5 ml) mixture. Then $CHCl_3$ (90 ml) and H2O (60 ml) were added. The organic layer was separated and evaporated to dryness. The solid residue was purified by size-exclusion chromatography with 2.5% of MeOH in $CHCl_3$. Finally, the obtained fraction was applied in CH_2Cl_2 to a silica column packed in CH_2Cl_2 and MeOH (0→5% of MeOH) mixtures. The residue was evaporated giving 83% (10 mg) of pure compound.

MALDI TOF MS: m/z calculated for $C_{69}H_{54}N_6NiO_4$ 1088.36, found 1089.38 (MH^+).

^{1}H NMR (600 MHz; $CDCl_3$; MeOD (15%); δ, ppm; J, Hz): 9.17 (d, 1H, ^3J = 7.9, H_{Phen}), 8.93 (d, 1H, ^3J = 8.7, H_{Phen}), 8.88 - 8.84 (m, 3H: 2$H_β$, H_{Phen}) 8.79 (d, 2H, ^3J = 4.9, $H_β$), 8.75 (d, 2H, ^3J = 4.9, $H_β$), 8.57 (d, 2H, ^3J = 4.9, $H_β$), 8.20 - 8.11 (m, 3H: 2$H_{Carboxy-Ph}$, H_{Phen}), 8.07 - 7.67 (m, 4H, H_{Ar}), 7.85 (t, 1H, ^3J = 7.5, H_{Phen}), 7.77 (t, 1H, ^3J = 7.8, H_{Phen}), 7.62 (t, 1H, ^3J = 7.8, H_{Phen}), 7.46 (t, 1H, ^3J = 7.6, H_{Phen}), 7.18 (d, 4H, ^3J = 7.7, H_{Ar}), 6.98 - 6.89 (m, 3H, H_{Bn}), 6.73 (d, 2H, ^3J = 7.4, H_{Bn}), 5.51 (s, 2H, H_{Bn}), 4.17 (t, 4H, ^3J = 6.5, H_{O-Bu}), 1.92 (quintet, 4H, ^3J = 6.8, H_{O-Bu}), 1.62 (sextet, 4H, ^3J = 7.4, H_{O-Bu}), 1.07 (t, 6H, ^3J = 7.4, H_{O-Bu}).

UV-Vis (CHCl3; λ_{max}/nm; log ε): 268 (4.80), 292 (4.35), 316 (4.21), 432 (5.18), 543 (4.12), 576 (3.70).

References

[1] Linstead RP, Doering WE. The Stereochemistry of Catalytic Hydrogenation. II. The Preparation of the Six Inactive Perhydrodiphenic 1 Acids. J Am Chem Soc 1942;64:1991–2003. https://doi.org/10.1021/ja01261a002.

[2] Birin KP, Gorbunova YG, Tsivadze AY. New approach for post-functionalization of meso-formylporphyrins. RSC Adv 2015;5:67242–6. https://doi.org/10.1039/C5RA13532J.

Spectral data of Ni-3

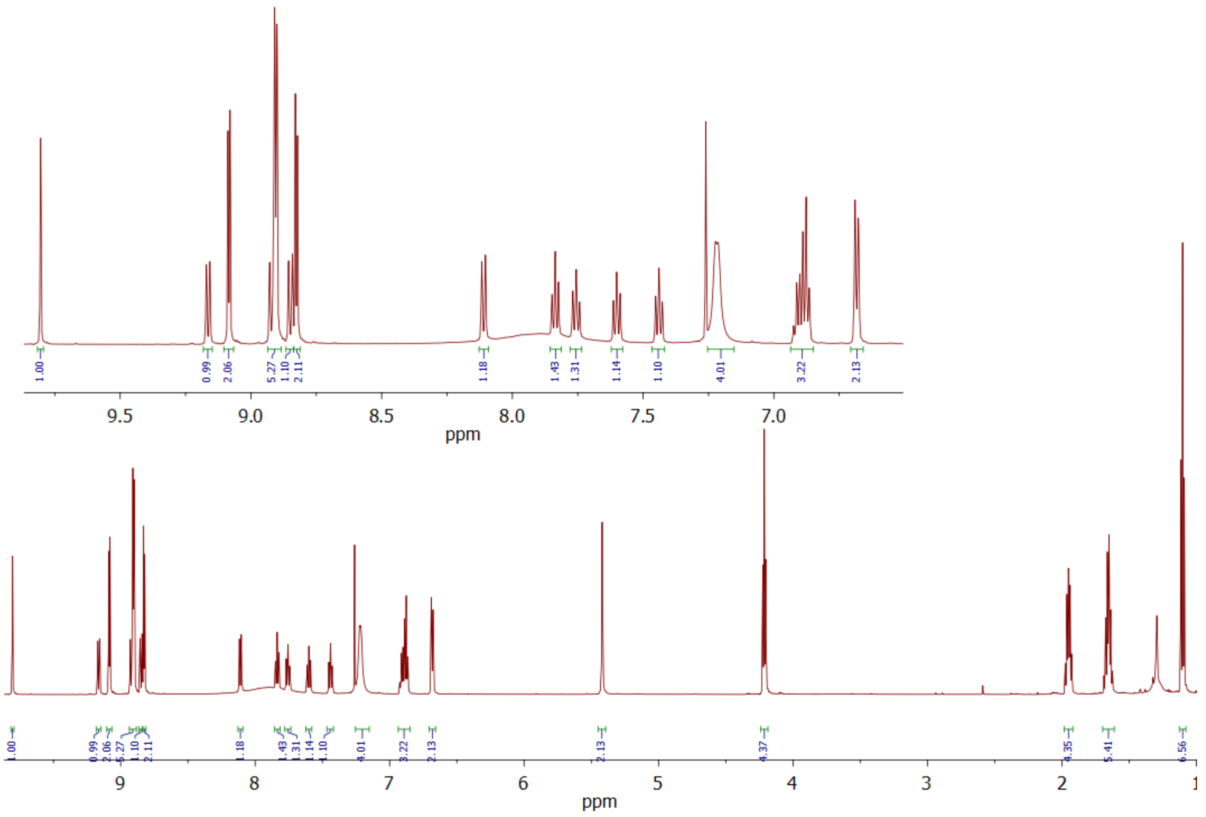

Figure S1. ^{1}H-NMR spectrum of **Ni-3** ($CDCl_3$).

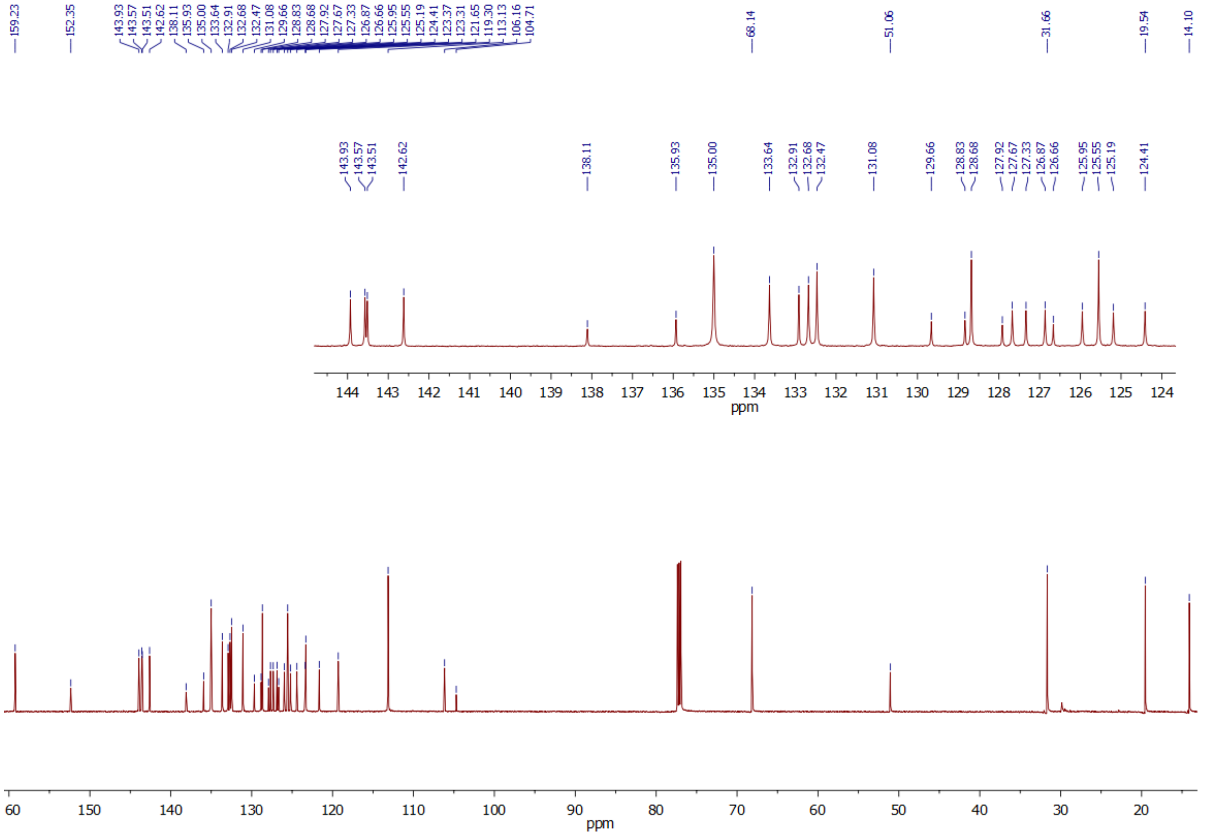

Figure S2. ^{13}C-NMR spectrum of **Ni-3** ($CDCl_3$).

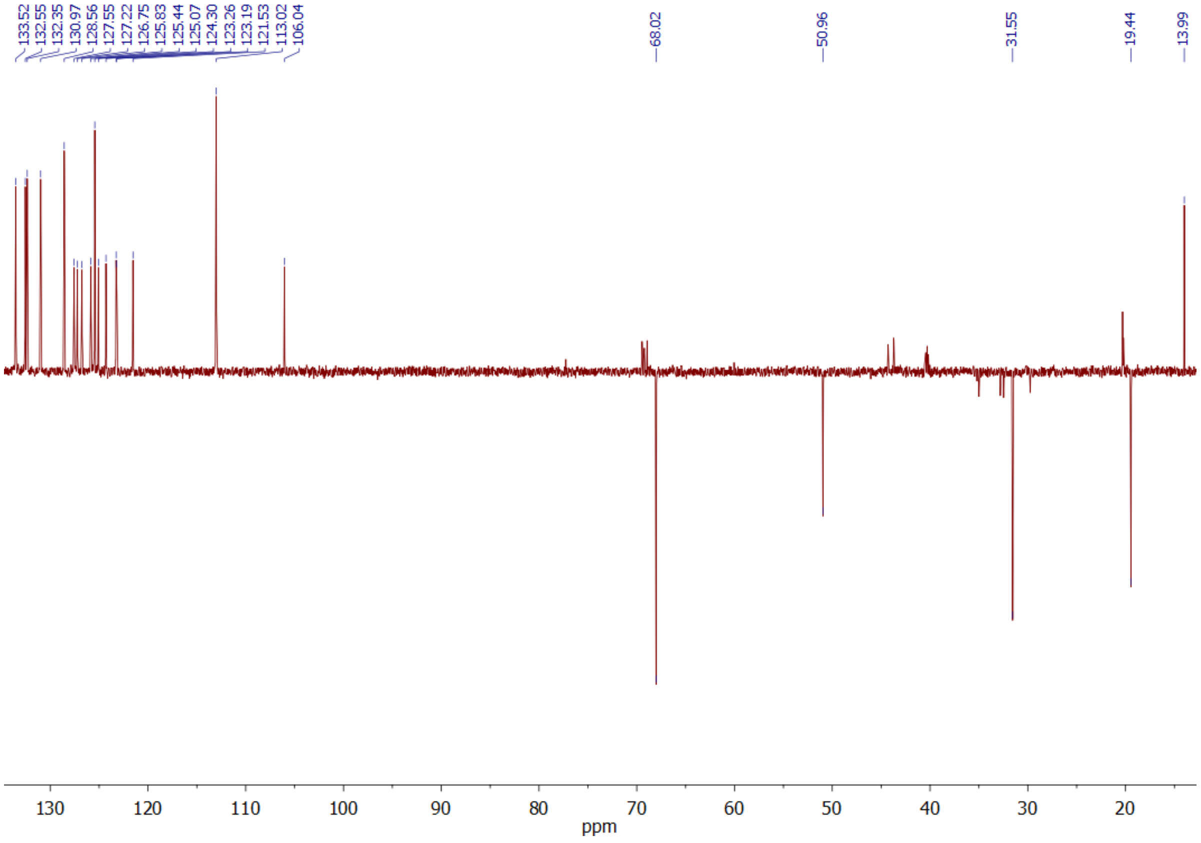

Figure S3. ^{13}C DEPT-135 spectrum of **Ni-3** ($CDCl_3$).

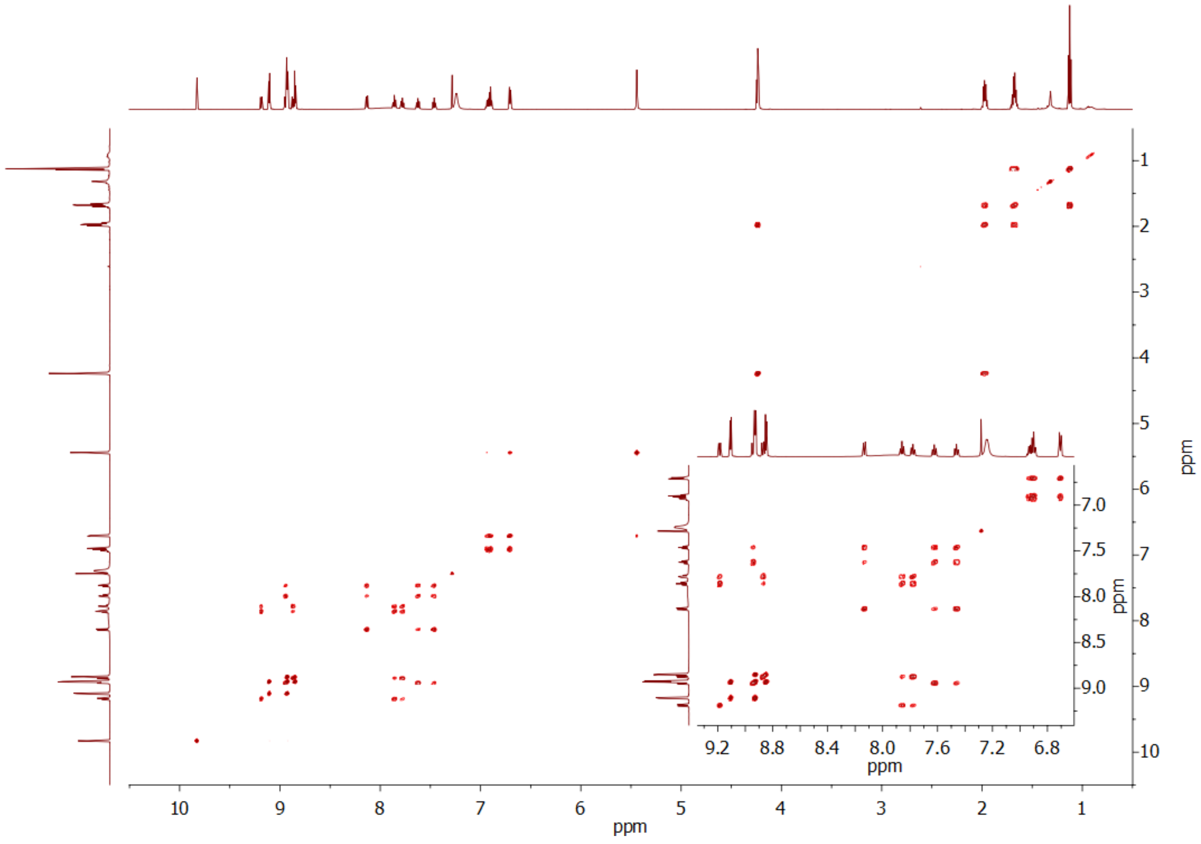

Figure S4. COSY spectrum of **Ni-3** ($CDCl_3$).

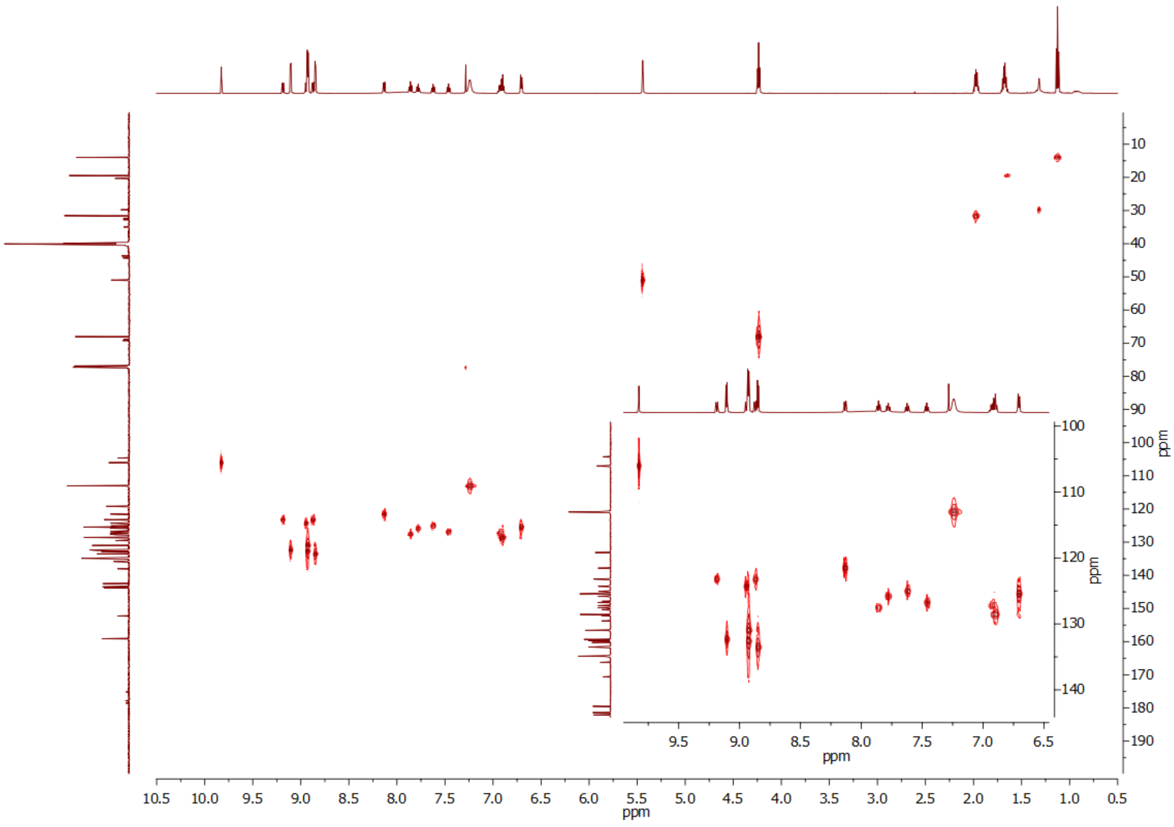

Figure S5. ^{1}H-^{13}C HSQC spectrum of **Ni-3** ($CDCl_3$).

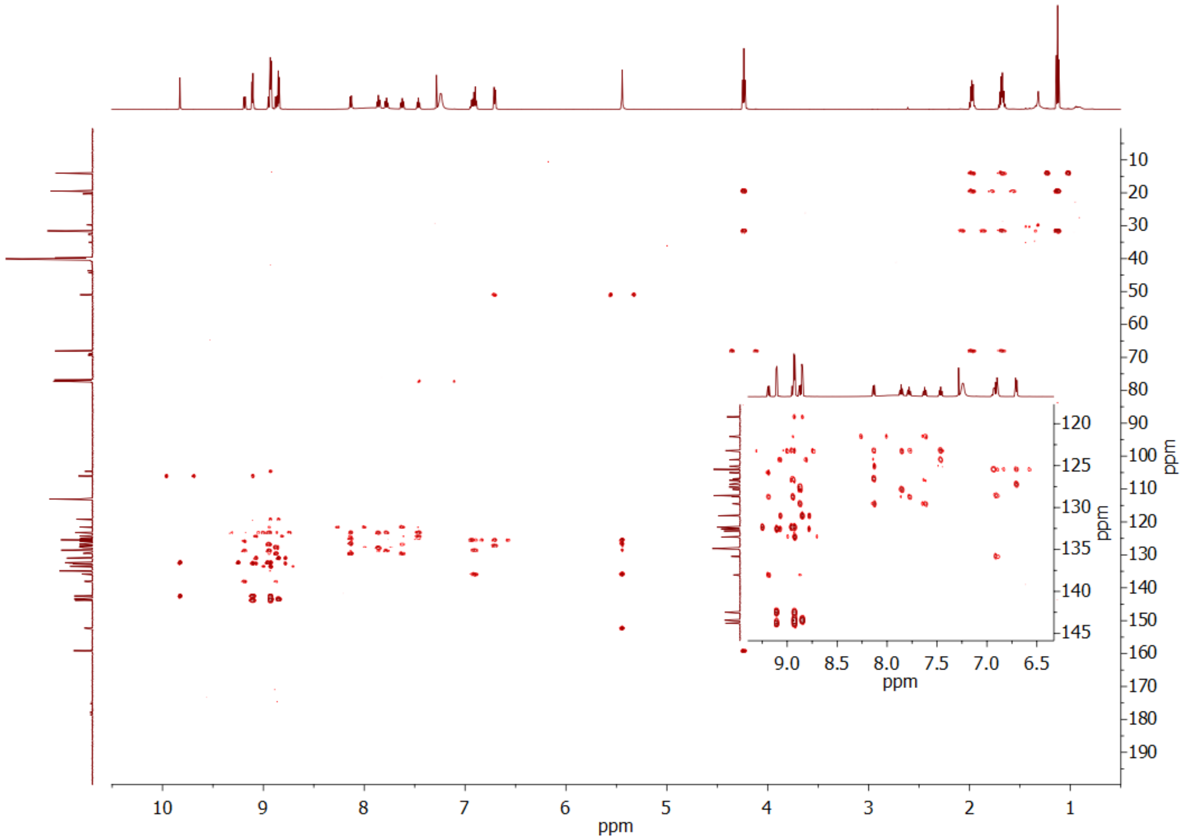

Figure S6. ^{1}H-^{13}C HMBC spectrum of **Ni-3** ($CDCl_3$).

Spectral data of Ni-4

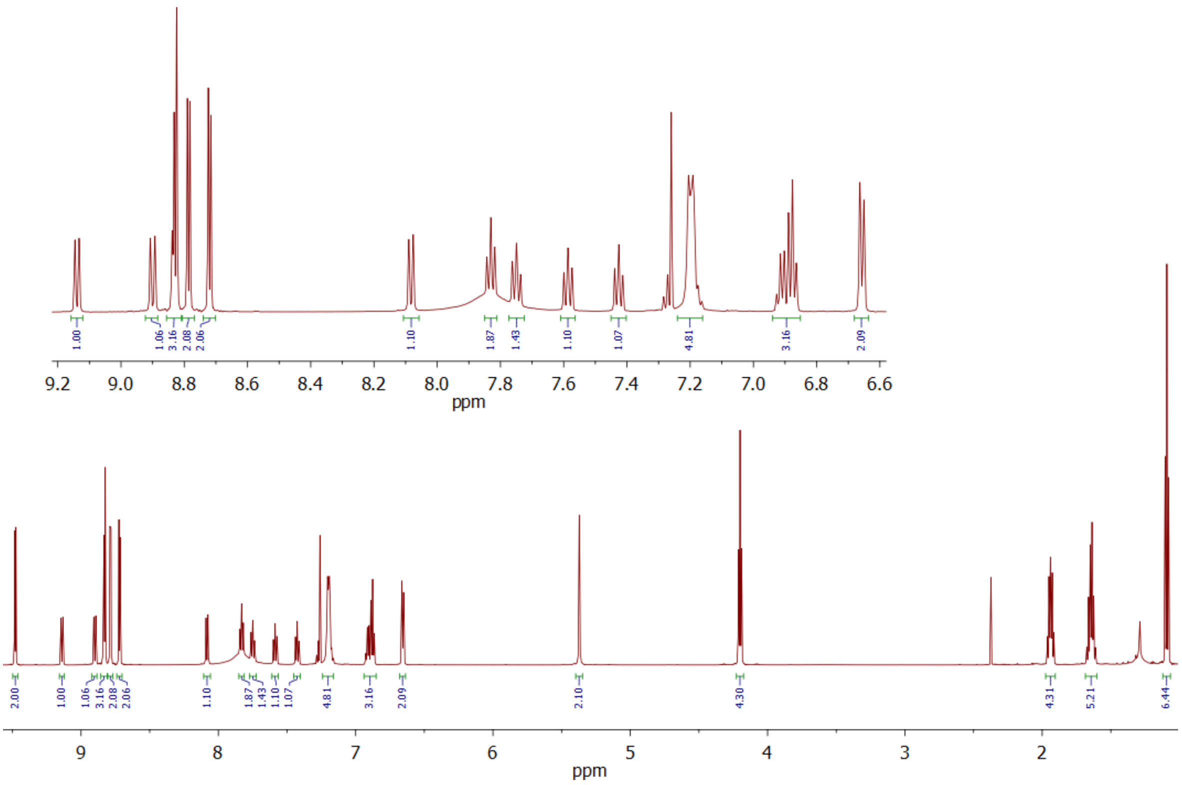

Figure S7. ^{1}H-NMR spectrum of **Ni-4** (CDCl3).

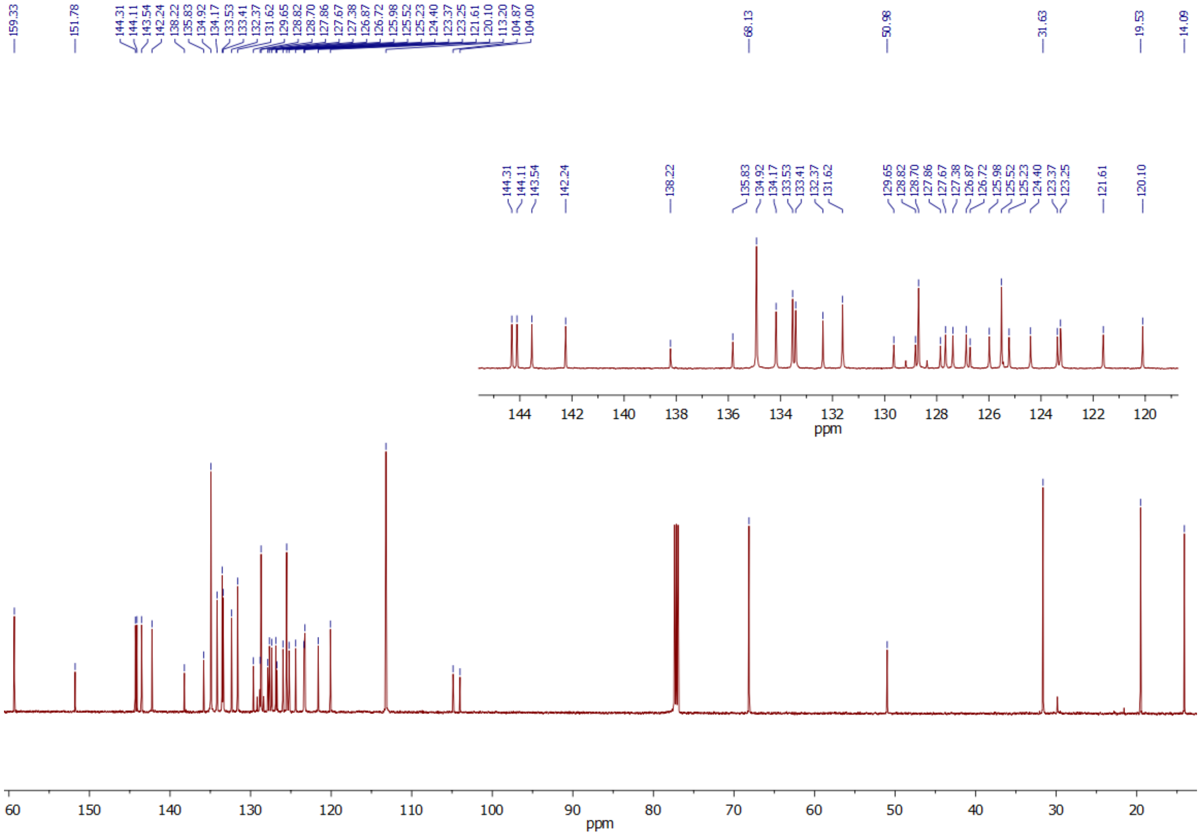

Figure S8. ^{13}C-NMR spectrum of **Ni-4** (CDCl3).

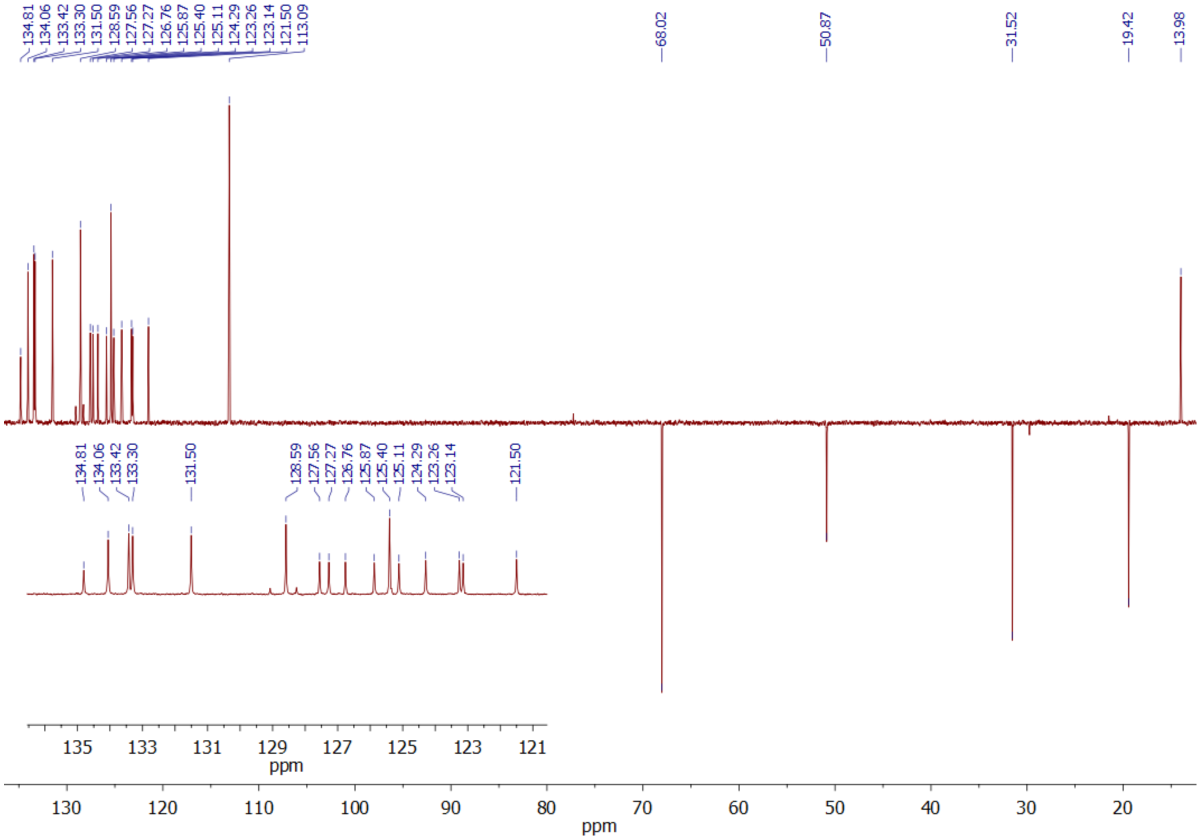

Figure S9. ^{13}C DEPT-135 spectrum of **Ni-4** ($CDCl_3$).

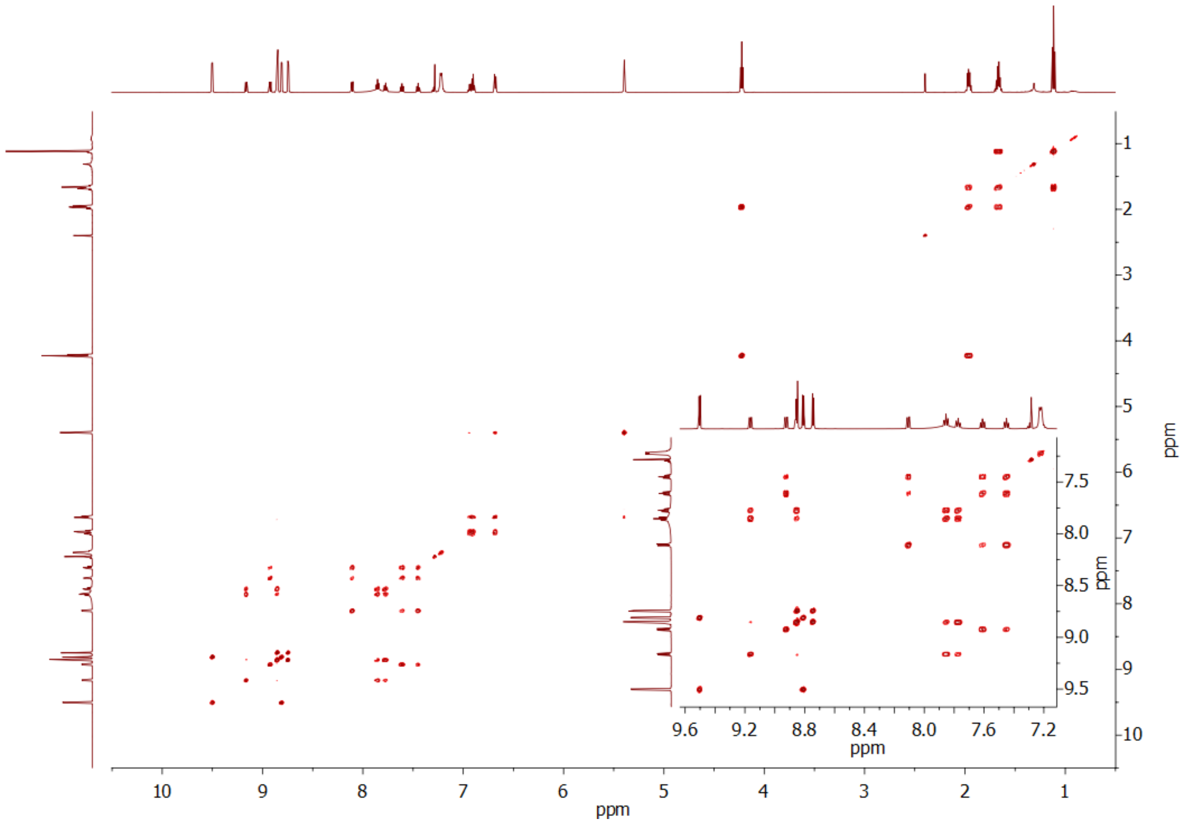

Figure S10. COSY spectrum of **Ni-4** ($CDCl_3$).

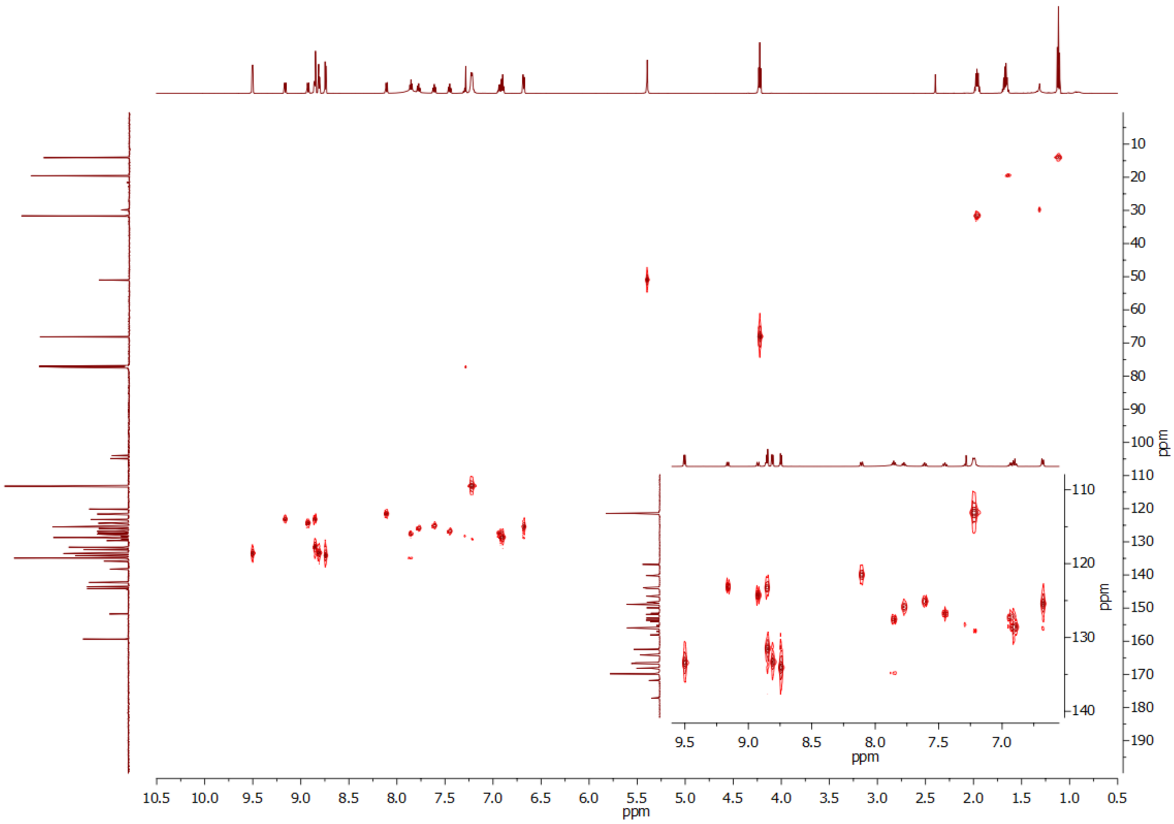

Figure S11. ^{1}H-^{13}C HSQC spectrum of **Ni-4** ($CDCl_3$).

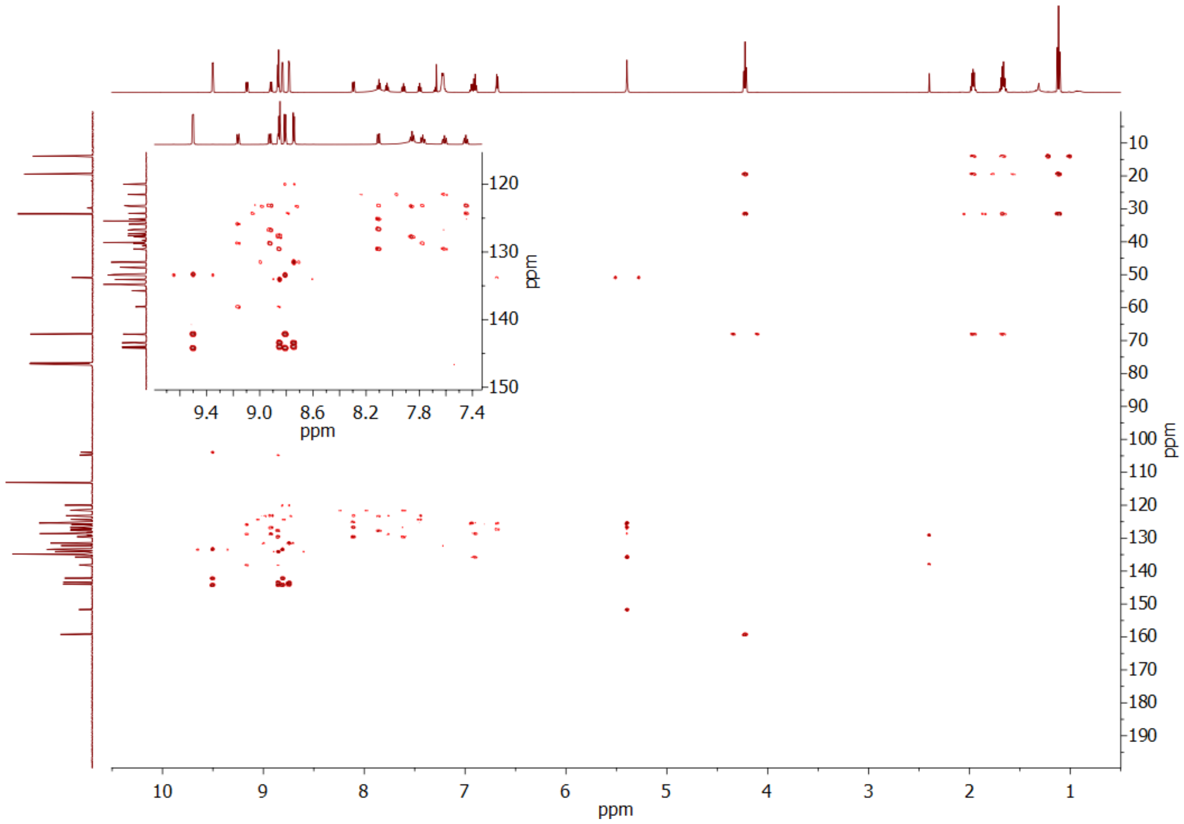

Figure S12. ^{1}H-^{13}C HMBC spectrum of **Ni-4** ($CDCl_3$).

Spectral data of Ni-5

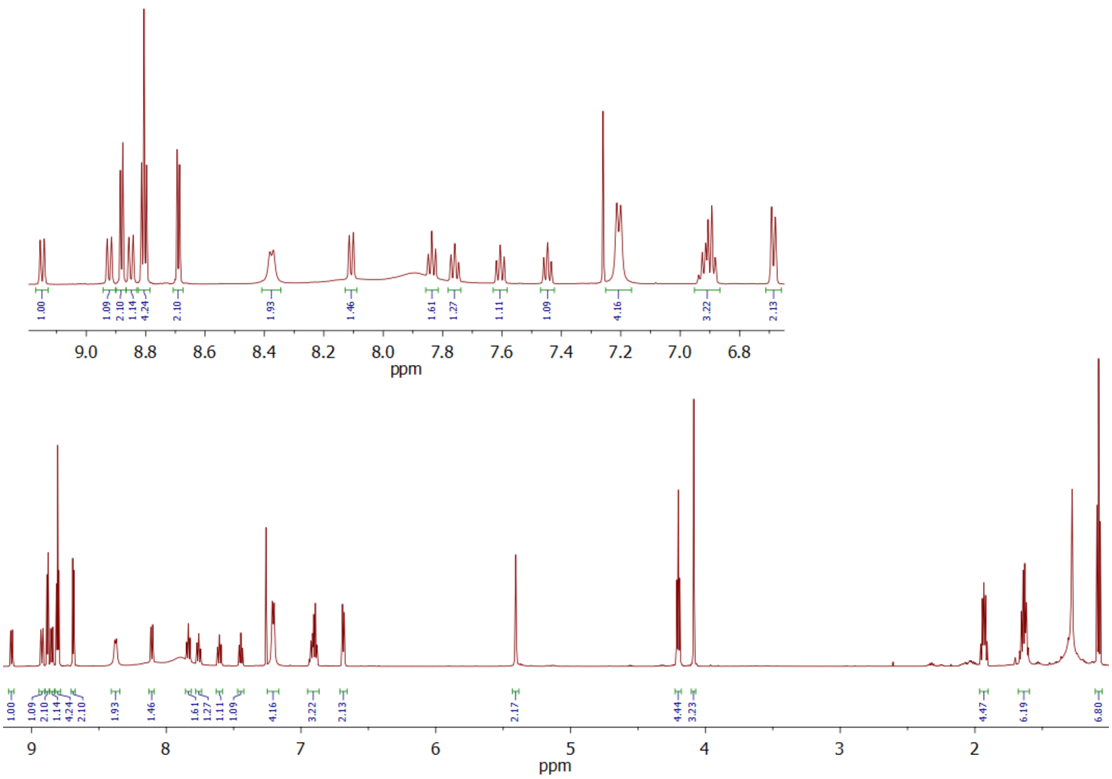

Figure S13. ^{1}H-NMR spectrum of **Ni-5** ($CDCl_3$).

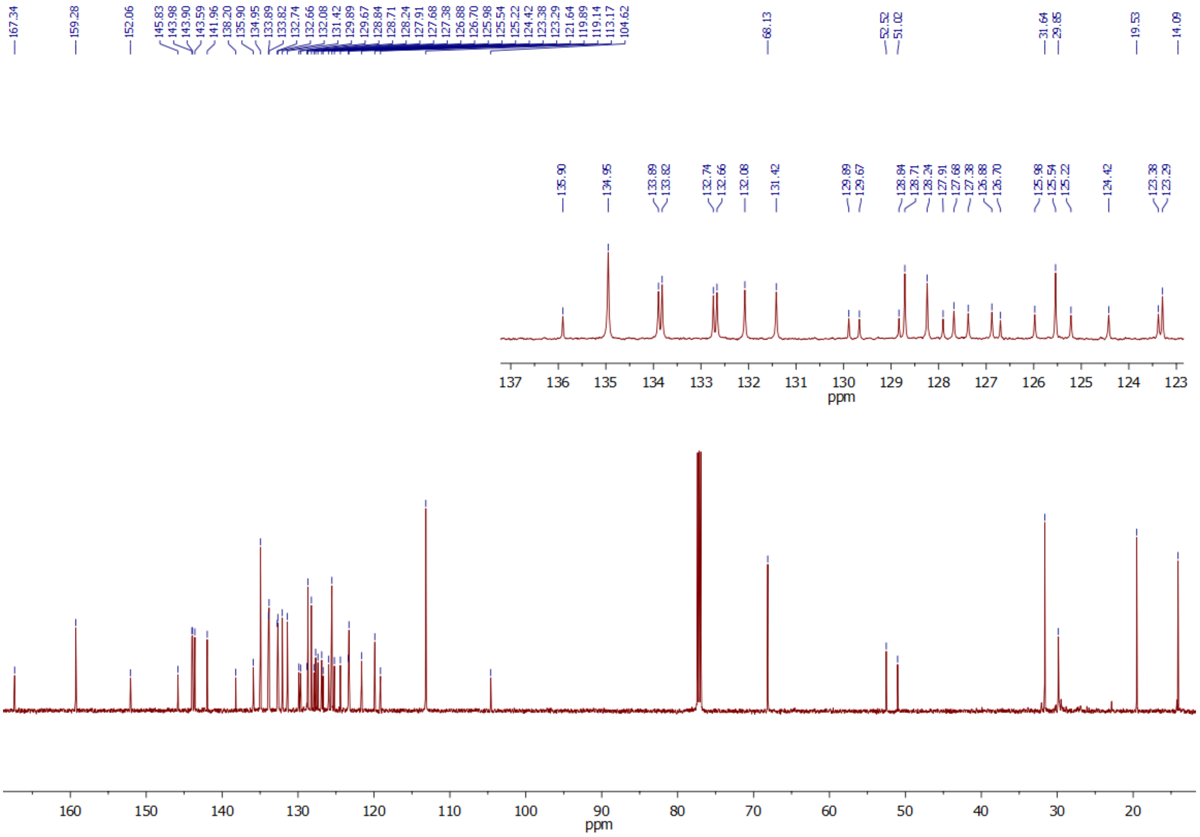

Figure S14. ^{13}C-NMR spectrum of **Ni-5** ($CDCl_3$).

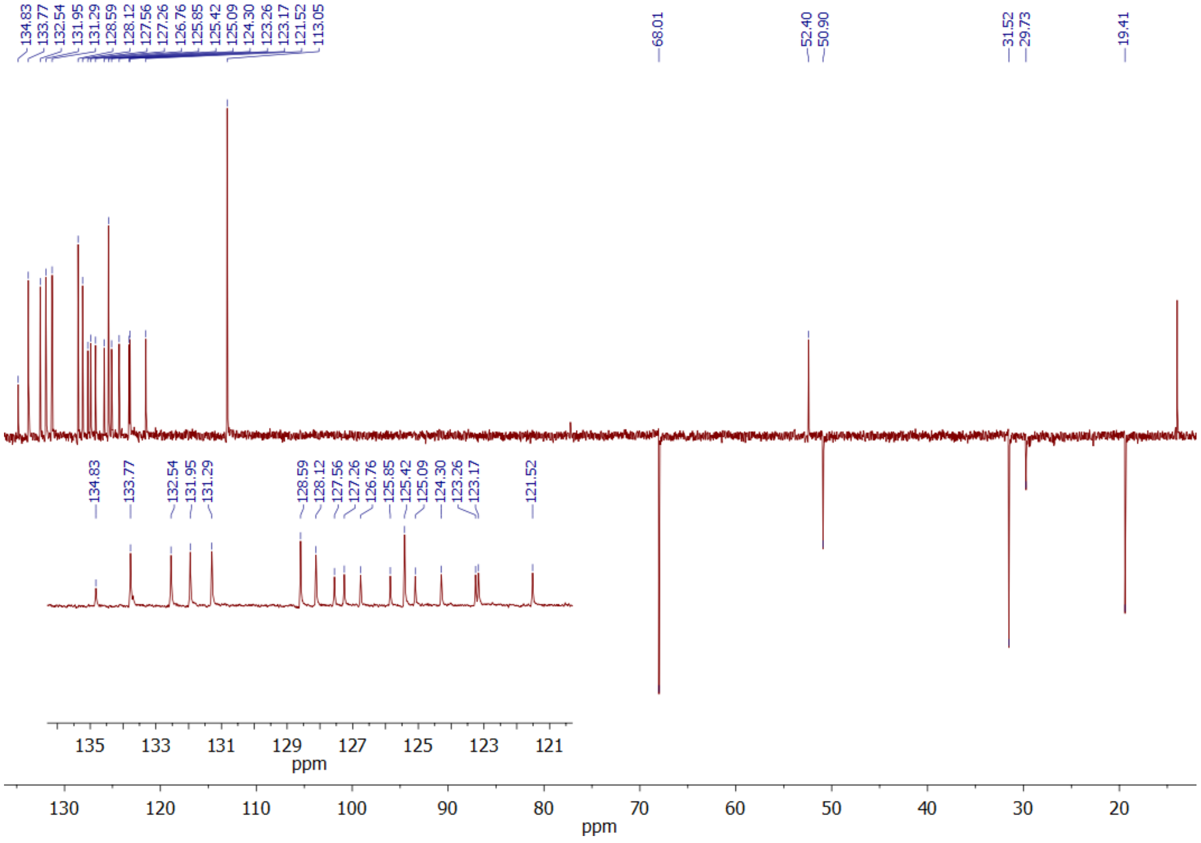

Figure S15. ^{13}C DEPT-135 spectrum of **Ni-5** ($CDCl_3$).

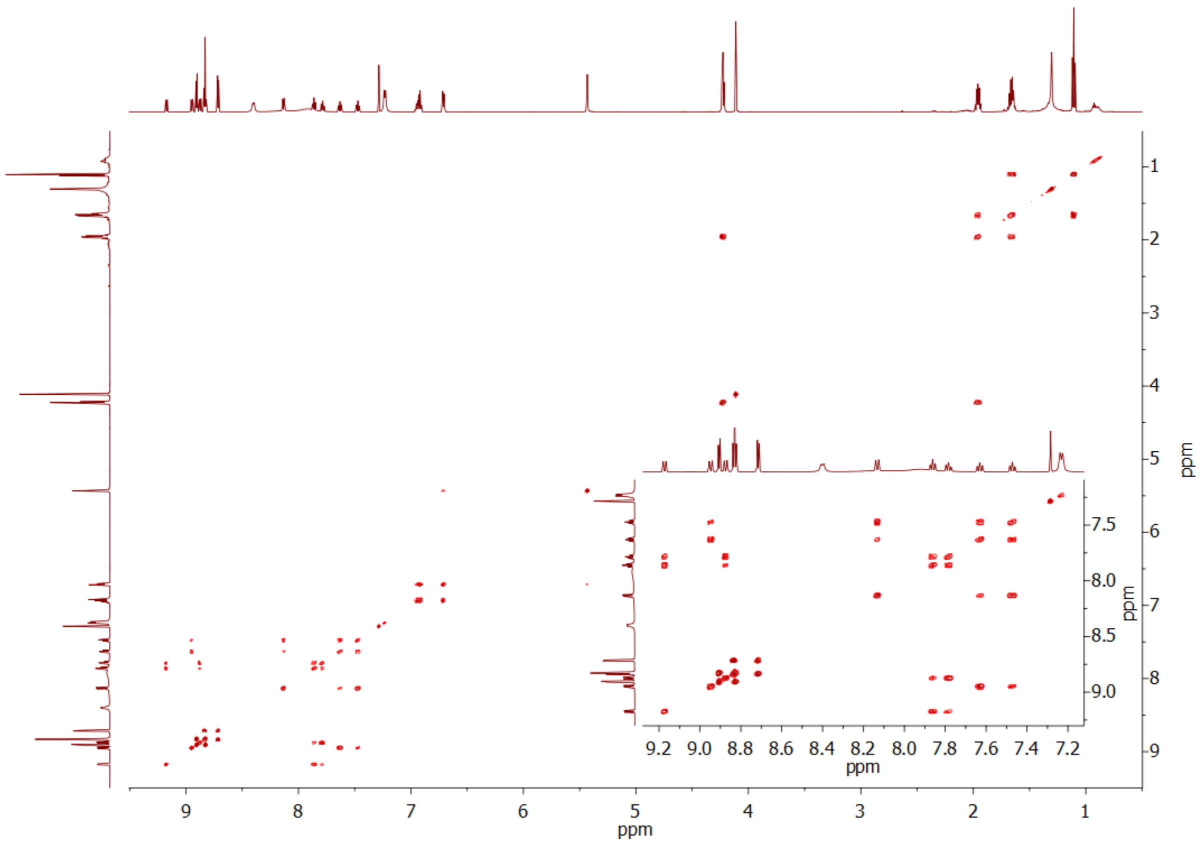

Figure S16. COSY spectrum of **Ni-5** ($CDCl_3$).

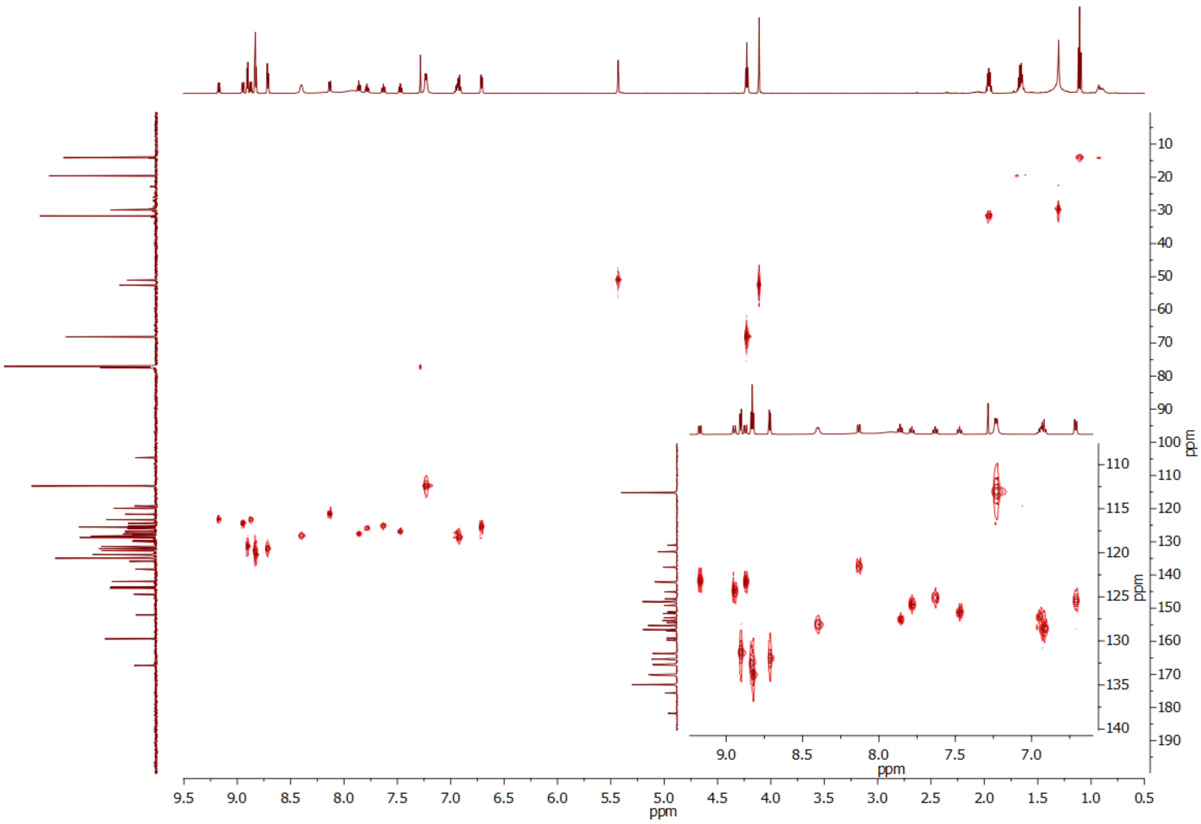

Figure S17. ^{1}H-^{13}C HSQC spectrum of **Ni-5** ($CDCl_3$).

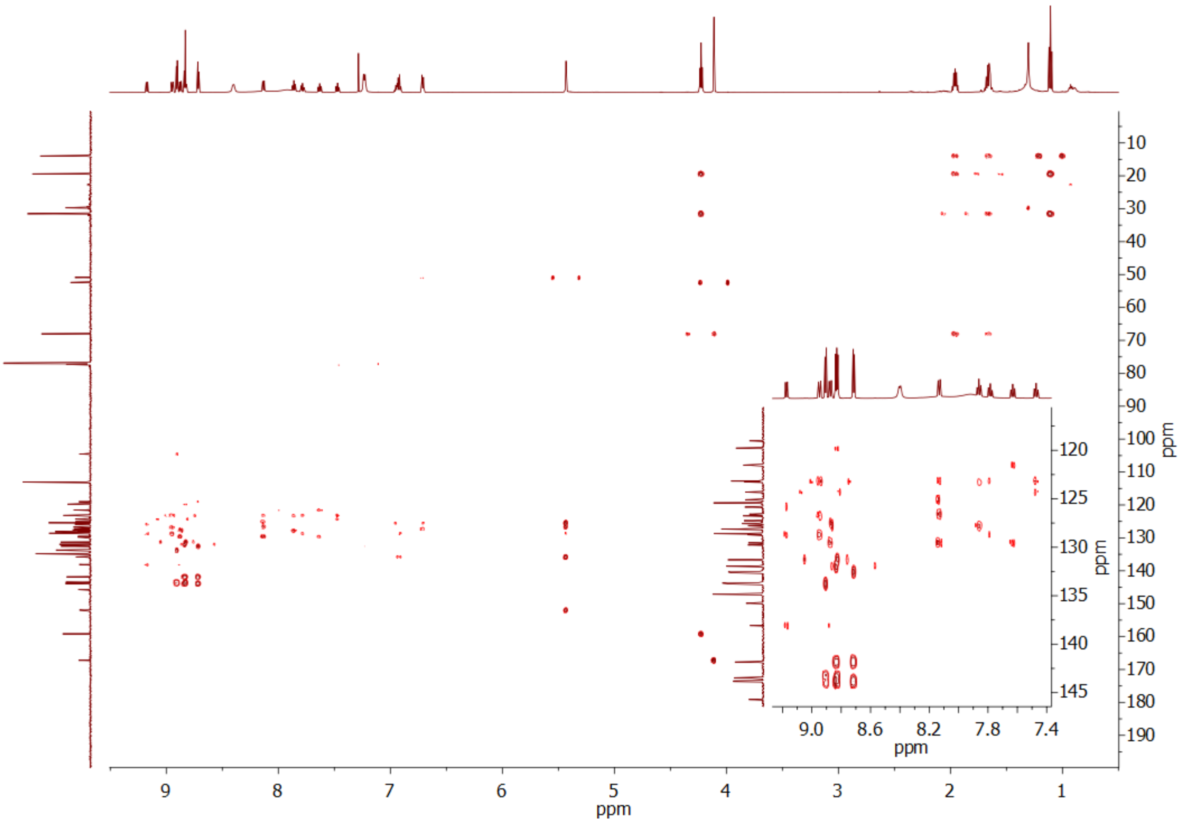

Figure S18. ^{1}H-^{13}C HMBC spectrum of **Ni-5** ($CDCl_3$).

Spectral data of Ni-6

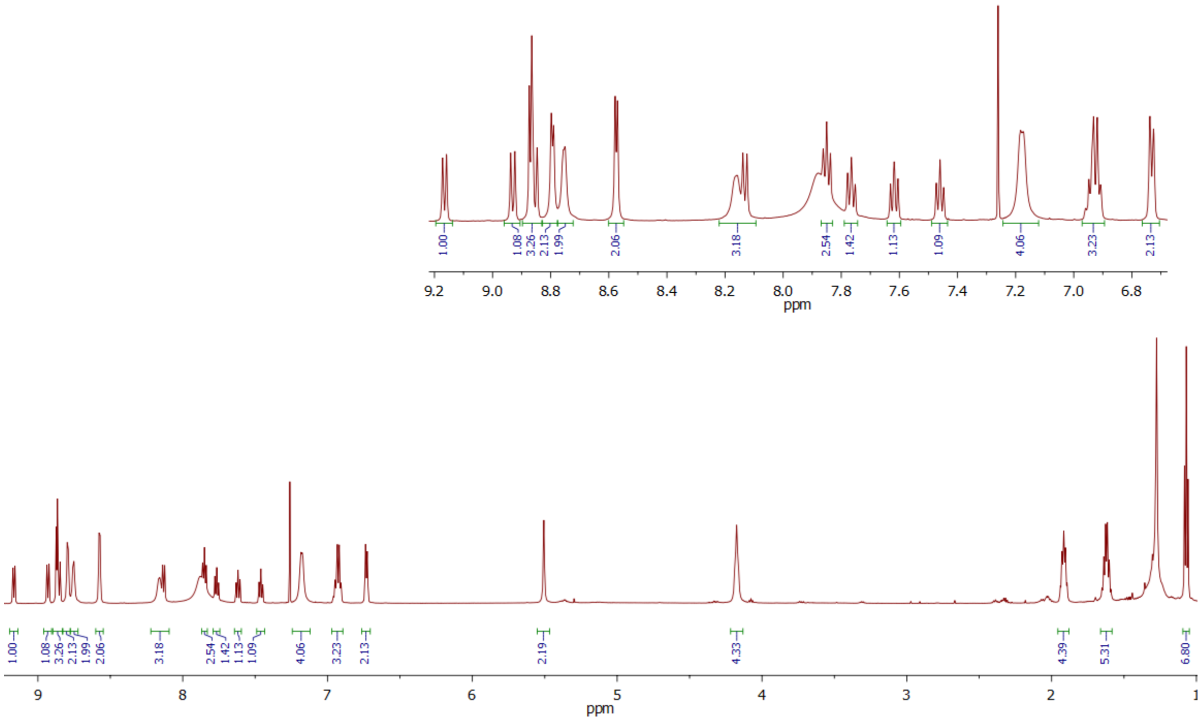

Figure S19. ^{1}H-NMR spectrum of **Ni-6** ($CDCl_3$).

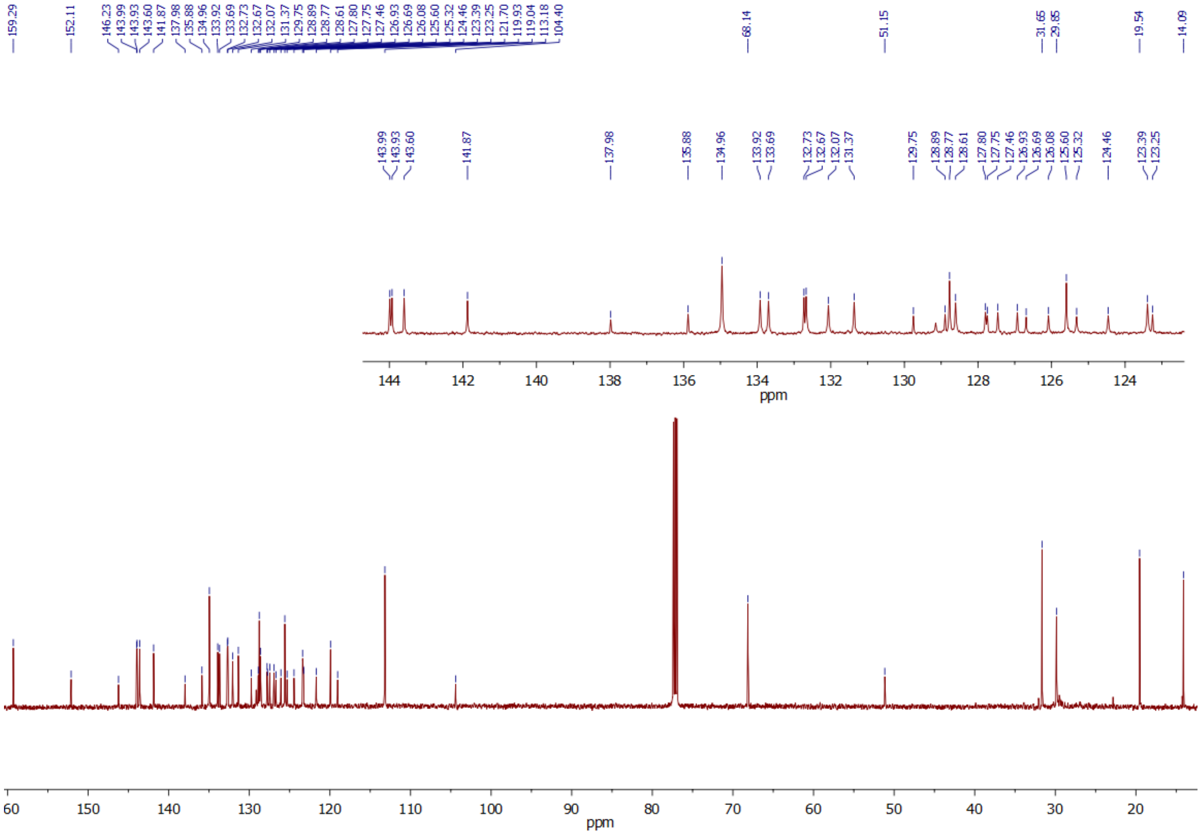

Figure S20. ^{13}C-NMR spectrum of **Ni-6** ($CDCl_3$).

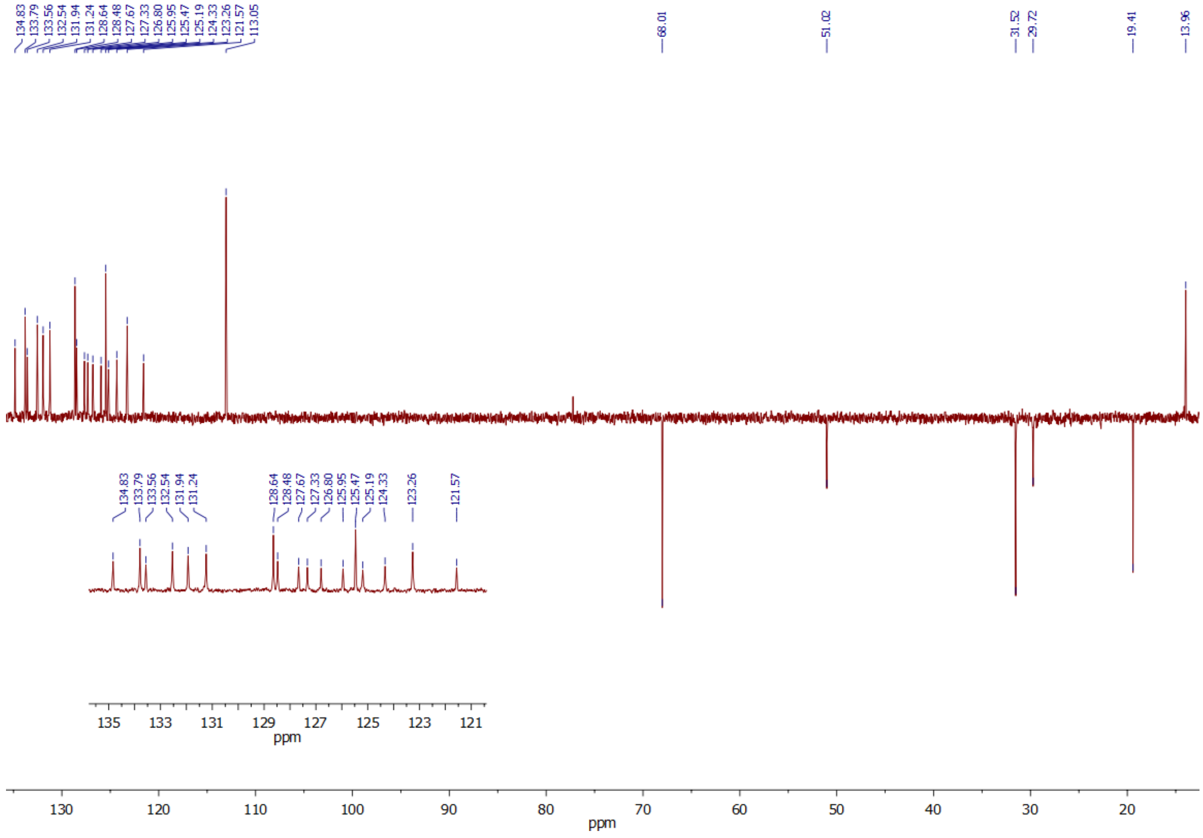

Figure S21. ^{13}C DEPT-135 spectrum of **Ni-6** ($CDCl_3$).

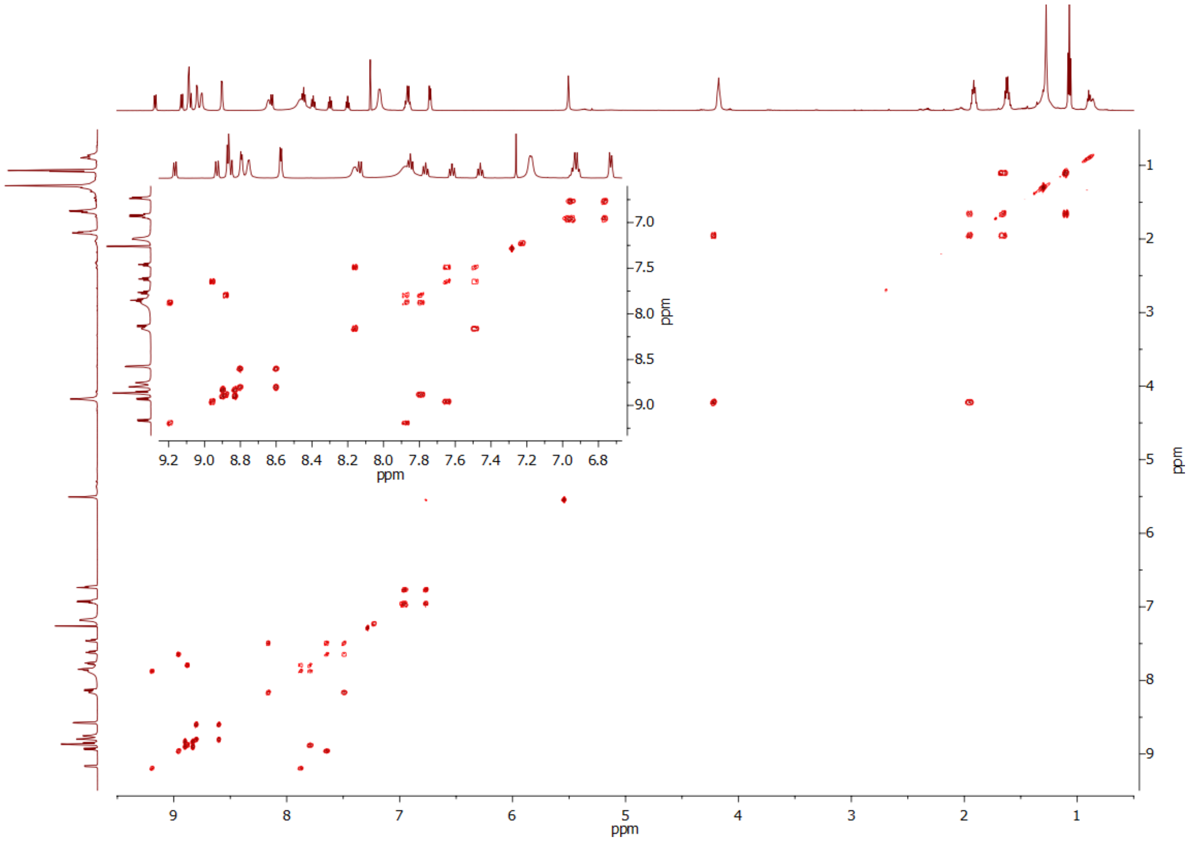

Figure S22. COSY spectrum of **Ni-6** ($CDCl_3$).

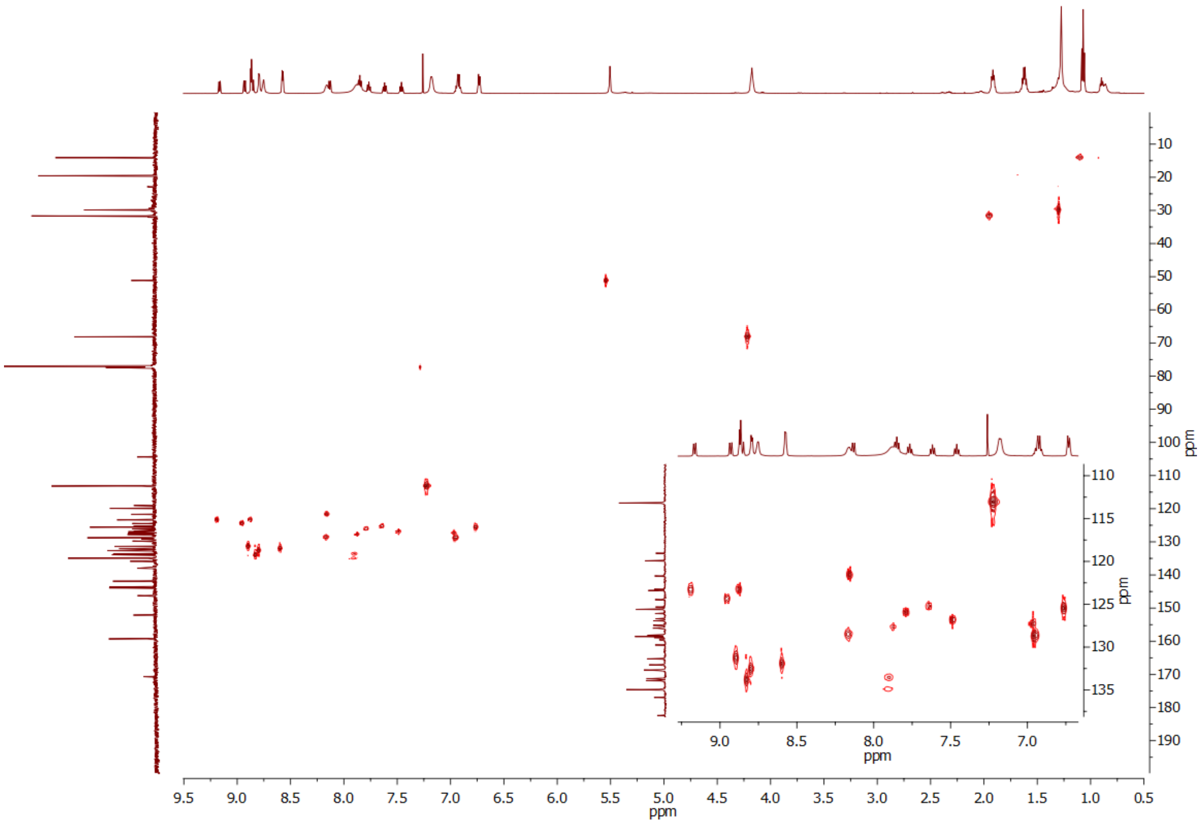

Figure S23. ^1H-^{13}C HSQC spectrum of **Ni-6** ($CDCl_3$).

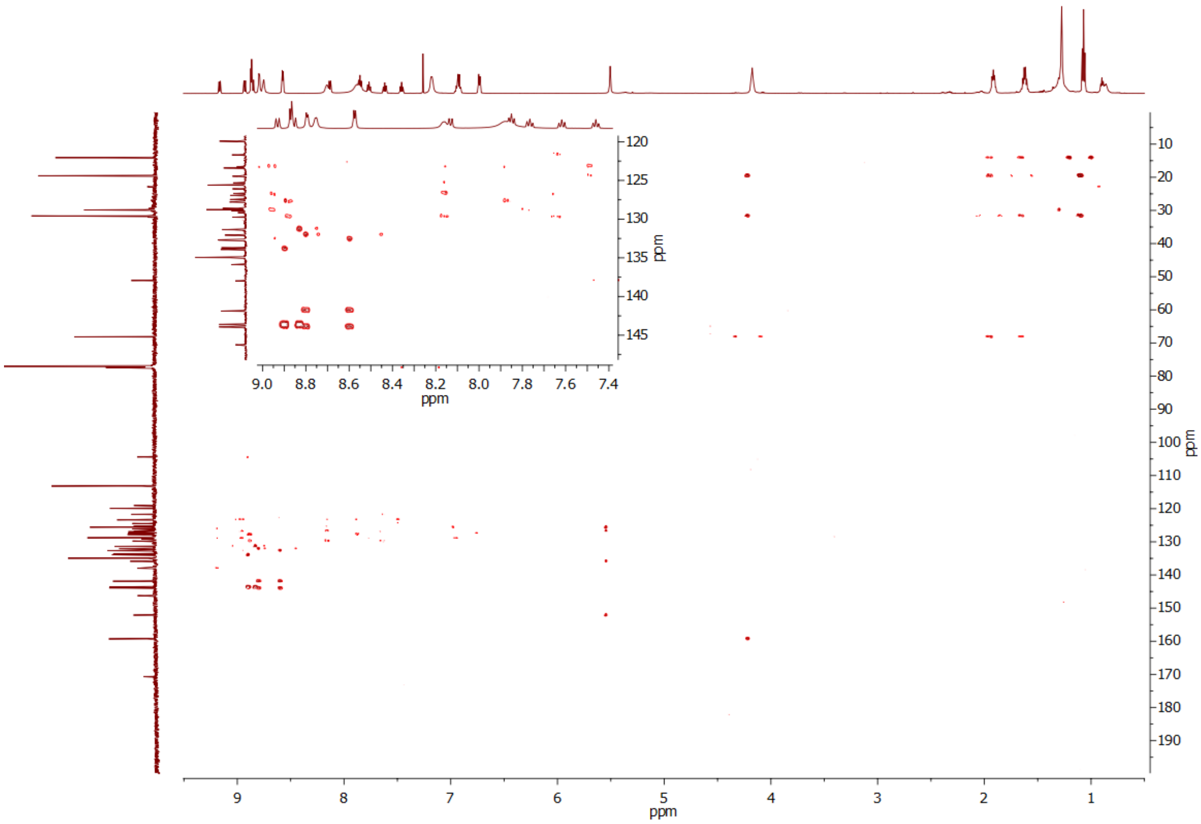

Figure S24. ^1H-^{13}C HMBC spectrum of **Ni-6** ($CDCl_3$).
